# Prognostic Value of Bronchoalveolar Lavage in Systemic Autoimmune Rheumatic Diseases-Associated Interstitial Lung Disease

**DOI:** 10.3390/jcm15124834

**Published:** 2026-06-22

**Authors:** Maximilian Robert Gysan, Kastriot Kastrati, Svitlana Pochepnia, Helmut Prosch, Antje Lehmann, Silvia Lee, Andreas Renner, Christina Bal, Anastasia Papaporfyriou, Christopher Milacek, Lukasz Antoniewicz, Seda Metekol, Markus Kramer, Lisa John, Zahra Kargarpour, Iris Aykara, Peter Weber, Karolina Anderle, Hans Peter Kiener, Michael Bonelli, Daniel Mrak, Daniel Aletaha, Ahmed El-Gazzar, Daniela Gompelmann, Marco Idzko, Helga Lechner-Radner

**Affiliations:** 1 Division of Pulmonology, Department of Internal Medicine II, Medical University of Vienna, 1090 Vienna, Austria; maximilian.gysan@meduniwien.ac.at (M.R.G.); antje.lehmann@meduniwien.ac.at (A.L.); silvia.lee@meduniwien.ac.at (S.L.); andreas.renner@meduniwien.ac.at (A.R.); christina.bal@meduniwien.ac.at (C.B.); anastasia.papaporfyriou@meduniwien.ac.at (A.P.); christopher.milacek@meduniwien.ac.at (C.M.); lukasz.antoniewicz@meduniwien.ac.at (L.A.); seda.metekol@meduniwien.ac.at (S.M.); markus.kramer@meduniwien.ac.at (M.K.); lisa.john@meduniwien.ac.at (L.J.); zahra.kargarpour@meduniwien.ac.at (Z.K.); ahmed.el-gazzar@meduniwien.ac.at (A.E.-G.); daniela.gompelmann@meduniwien.ac.at (D.G.); 2 Division of Rheumatology, Department of Internal Medicine III, Medical University of Vienna, 1090 Vienna, Austria; kastriot.kastrati@meduniwien.ac.at (K.K.); iris.aykara@gmail.com (I.A.); peter.weber@meduniwien.ac.at (P.W.); karolina.anderle@meduniwien.ac.at (K.A.); hans.kiener@meduniwien.ac.at (H.P.K.); michael.bonelli@meduniwien.ac.at (M.B.); daniel.mrak@meduniwien.ac.at (D.M.); daniel.aletaha@meduniwien.ac.at (D.A.); helga.lechner-radner@meduniwien.ac.at (H.L.-R.); 3 Department of Biomedical Imaging and Image-Guided Therapy, Medical University of Vienna, 1090 Vienna, Austria; svitlana.pochepnia@meduniwien.ac.at (S.P.); helmut.prosch@meduniwien.ac.at (H.P.); 4 Department of Medicine II, Lower Austrian Centre for Rheumatology, Karl Landsteiner Institute for Clinical Rheumatology, State Hospital Stockerau, 2000 Stockerau, Austria

**Keywords:** interstitial fibrosis, systemic autoimmune rheumatic diseases-associated interstitial lung disease, bronchoscopy, bronchoalveolar lavage

## Abstract

**Background:** Systemic autoimmune rheumatic diseases-associated interstitial lung disease (SARD-ILD) presents with varied disease courses, emphasizing the need for reliable predictors of progression. The prognostic utility of bronchoalveolar lavage (BAL) in SARD-ILD remains underexplored. The objective of this study was to evaluate the role of BAL fluid lymphocyte count in predicting disease progression in patients with SARD-ILD. **Methods:** This observational study included patients with SARD-ILD undergoing BAL as part of their diagnostic workup. Disease progression was defined as either Forced vital capacity (FVC) decrease >10%, two out of the following three criteria within two years: FVC decrease of 5–10%, worsening symptoms, increased fibrosis on imaging, or any of the following: escalation of treatment, Interstitial lung disease (ILD) exacerbation, lung transplantation, or disease-specific mortality. Logistic regression identified predictors of progression. Time-to-progression was assessed using Kaplan–Meier survival curves. The optimal BAL lymphocyte threshold for predicting progression was identified using the Youden Index and the Wilcoxon method. **Results:** We identified 89 patients, of whom 30 (33.7%) had progressive disease. Progressors had a significantly higher BAL lymphocyte count compared to non-progressors (31.6 ± 24.8% vs. 14.3 ± 16.5%, *p* < 0.001). BAL lymphocyte proportion was significantly and independently associated with disease progression (odds ratio, 1.05; 95% confidence interval 1.02–1.07; *p* < 0.01). A lymphocyte count above 9 percent was associated with a markedly increased risk of disease progression (odds ratio, 13.14; 95% confidence interval, 4.20–51.98; *p* < 0.01). **Conclusions:** BAL lymphocyte count was associated with a higher likelihood of progression in SARD-ILD. BAL assessment may help identify patients at increased risk of disease progression. However, these findings should be considered exploratory and require validation in larger prospective studies and across individual SARD-ILD subtypes.

## 1. Introduction

Interstitial lung disease (ILD) comprises a heterogenous group of diffuse parenchymal lung disorders which are defined by specific clinical, histopathological and radiological features. ILD often occurs idiopathically, and may be caused by environmental exposure or by an underlying systemic autoimmune disease including systemic sclerosis (SSc), rheumatoid arthritis (RA), idiopathic inflammatory myositis (IIM), Sjögren’s syndrome (SS), mixed connective-tissue disease (MCTD) and systemic lupus erythematosus (SLE). Systemic autoimmune rheumatic diseases (SARD) frequently lead to interstitial lung disease [1,2,3,4,5]. Particularly in RA and SSc, ILD is linked to substantial morbidity and mortality [6,7]. The clinical presentation of SARD-ILD is characterized by exertional dyspnoea, dry cough and, less frequently, finger clubbing [8]. Treatment strategies in SARD-ILD include immunosuppressive and antifibrotic agents [9,10,11].

The diagnostic evaluation of interstitial lung disease comprises a comprehensive medical history, pulmonary function tests (PFT) including body plethysmography, the assessment of diffusion capacity, blood works including serologic studies and a high-resolution computed tomography (HRCT). Cases should then be reviewed in a multidisciplinary discussion (MDD). Depending on the predominant HRCT pattern, further diagnostic assessment includes bronchoscopy with bronchoalveolar lavage (BAL) and transbronchial lung cryobiopsy (TBLC) [12]. The predominant histopathological and HRCT pattern in SARD-ILD is non-specific interstitial pneumonia (NSIP) with the exception of RA-ILD, where usual interstitial pneumonia (UIP) prevails [13,14,15,16]. Subclinical interstitial lung abnormalities can also be present on HRCT. Disease trajectories in SARD-ILD vary, as demonstrated for SSc and RA [17,18]. Predicting disease progression for individual patients is essential to initiate timely treatment and estimate prognosis. In recent years, efforts have been made to define valid criteria for disease progression in ILD other than idiopathic pulmonary fibrosis, exemplified by the INBUILD trial and the ATS/ERS/JRS/ALAT clinical practice guideline for progressive pulmonary fibrosis [9,19]. Bronchoalveolar lavage (BAL) is a well-established tool in the diagnostic evaluation of interstitial lung disease [20]. Its primary diagnostic role is to exclude alternative conditions such as infection, malignancy, and diffuse alveolar hemorrhage. While certain BAL differential cell count patterns may be highly suggestive of particular diagnoses, such as eosinophilic pneumonia, BAL findings are generally not disease-specific and must be interpreted alongside clinical, radiological, and histopathological findings to reach a diagnosis. In contrast, the prognostic role of BAL has been investigated less extensively with a focus on idiopathic interstitial pneumonias and exacerbation [21,22], whereas its prognostic role in SARD-ILD remains underexplored. Identifying patients at risk of worsening disease presents a significant clinical challenge. Previous studies have demonstrated the prevalence of lymphocytes in bronchoalveolar lavage fluid (BALF) samples of patients with SARD-ILD [23,24,25,26]. Increased BAL lymphocyte proportions have been described across several SARD-ILD subtypes, suggesting a potentially shared role of lymphocytes in the alveolar compartment in promoting inflammation and subsequent fibrosis. Pooling different SARD-ILD entities, therefore, allowed us to investigate the prognostic significance of BAL lymphocytosis across a broader spectrum of autoimmune-associated interstitial lung diseases. With this study, we aim to investigate the role of lymphocytes in the alveolar compartment of patients with SARD-ILD and assess their potential for distinguishing between progressive and non-progressive disease.

## 2. Materials and Methods

### 2.1. Study Design and Ethical Approval

In this observational, monocentric study, we consecutively included patients with rheumatic and musculoskeletal diseases (RMDs), diagnosed according to disease-specific classification criteria [27,28,29,30,31,32], who underwent bronchoalveolar lavage (BAL) between July 2018 and October 2023 for newly diagnosed or progressive interstitial lung disease (ILD). These patients were in treatment at or referred to the Division of Pulmonology and Rheumatology at the Medical University of Vienna, a tertiary centre in Austria, and underwent BAL as part of their diagnostic workup and were followed up for two years. The decision to perform BAL was ultimately made by the treating clinician, primarily in response to new onset or progressive ILD. Inclusion criteria consisted of the following: (a) age of 18 years or older and (b) availability of clinical data, including pulmonary function tests (PFTs), treatment information, laboratory parameters, and imaging results from high-resolution computed tomography (HRCT) of the chest. The following exclusion criteria were applied: (a) non-SARD-ILD-related pulmonary disease and (b) missing follow-up data necessary to determine pulmonary disease progression. The methodology and reporting of this study adhere to the Strengthening the Reporting of Observational Studies in Epidemiology (STROBE) guidelines. This study was approved by the Ethics Committee of the Medical University of Vienna (identification no. 1127/2024).

### 2.2. Assessment and Outcome Measures

With regard to patients with SARD-ILD, clinical data were obtained from our prospective ILD registry and supplemented with information from electronic medical records (EMR) including the following: severity of respiratory symptoms as determined by the New York Heart Association (NYHA) classification tool [33], serum laboratory parameters (such as acute phase reactants, including C-reactive protein (CRP), absolute and relative counts of peripheral leukocytes, lymphocytes, and monocytes), imaging results from HRCT of the chest, PFTs, and the diffusing capacity of the lung for carbon monoxide (DLCO) adjusted for hemoglobin. Additionally, details regarding the initiation or escalation of immunosuppressive or antifibrotic therapies within a two-year observational period were documented. For all enrolled patients with SARD-ILD, PFT and HRCT results closest to the time of bronchoscopy and BAL were assessed. In accordance with recommendations [34], PFTs were systematically repeated every three to six months, while HRCT imaging was performed at the discretion of the treating clinician.

Patients were classified into ‘progressors’ and ‘non-progressors’ based on an adapted version of the INBUILD criteria for progressive pulmonary disease within a two-year period, absent any alternative explanation.

Progression was defined as either: (a) a relative decline in the FVC of at least 10% of the predicted value, (b) a relative decrease in FVC ranging from 5% to less than 10% of the predicted value accompanied by worsening respiratory symptoms or increased fibrosis on high-resolution CT or (c) a combination of worsening respiratory symptoms and increased fibrosis extent [9]. This was supplemented by an additional criterion: (d) and/or the need for intensification of immunosuppressive and/or antifibrotic therapy due to worsening respiratory symptoms, including hospitalization for respiratory distress or ILD exacerbation and/or lung transplantation, and/or death attributable to ILD.

Therapy intensification included adding systemic glucocorticoids (GC), doubling GC dosage, introducing immunosuppressive/antifibrotic agents, or initiating long-term oxygen therapy (LTOT). Therapy escalation occurring within six months of BAL in therapy-naive patients was excluded to avoid misclassification as progressors. Non-progression was defined by the absence of these criteria. BAL proceedings and HRCT analysis details are available in the Online Supplementary Materials.

### 2.3. Statistical Analysis

Statistical analyses were performed using R version 4.2.2 (R Development Core Team, Vienna, Austria). Continuous variables were expressed as mean with standard deviation (SD) or median with interquartile range (IQR), depending on the distribution assessed by the Shapiro–Wilk test. Categorical variables were reported as absolute frequencies and percentages. Comparisons between progressors and non-progressors were made using the Wilcoxon rank sum test for continuous variables and Chi-square or Fisher’s exact test for categorical variables. A 5% significance level was applied. Binary logistic regression assessed the predictive value of BAL lymphocyte proportion with clinical, radiological, and PFT parameters. We employed a forward stepwise approach, starting with age, FVC and extent of disease on HRCT as foundational variables. From this baseline model, additional clinically relevant variables (sex, disease duration of ILD, SARD diagnosis, DLCO, HRCT pattern, and treatment regimen) were sequentially added to refine the model. For each iteration, we compared models with and without the inclusion of BAL lymphocyte proportion to evaluate its independent contribution to predicting disease progression. In a separate model, we examined the impact of HRCT patterns independent of SARD diagnosis, as certain diagnoses, like Sjögren’s syndrome, are associated with specific patterns such as LIP. This allowed us to assess the influence of HRCT pattern on disease progression without confounding it with the underlying SARD diagnosis. The treatment regimen was modelled using separate binary variables for each therapy with presumed pulmonary efficacy. Model performance was evaluated using Nagelkerke’s pseudo-R-squared values and the Akaike Information Criterion (AIC). Given the relatively small sample size and limited number of progression events per endpoint, additional sensitivity analyses were performed using Firth’s penalized maximum likelihood logistic regression to reduce small-sample bias and potential model overfitting. Additionally, time-to-progression was assessed using Kaplan–Meier survival curves from the time of BAL performance (baseline/time zero), stratified by lymphocyte percentage in BAL. Initially, patients were grouped based on a BAL lymphocyte proportion of >15% versus ≤15% in accordance with guideline-based thresholds [35]. Optimal BAL lymphocyte cut-off values were determined using AUC sensitivity/specificity analyses (Youden Index) and the Wilcoxon method. As these analyses were performed in an observational cohort without external validation, all identified thresholds and diagnostic performance measures should be considered exploratory and hypothesis-generating.

## 3. Results

### 3.1. Patient Characteristics

We included a total of 89 patients with an established diagnosis of SARD-ILD in the analysis. 49 patients (55.1%) underwent BAL as part of the diagnostic evaluation for newly diagnosed ILD, while 40 patients (44.9%) underwent BAL due to pulmonary disease progression. The cohort encompassed a broad spectrum of SARD diagnoses, including IIM (*n* = 15), SSc (*n* = 21), RA (*n* = 15), MCTD (*n* = 23), SS (*n* = 8), and others (IPAF, *n* = 7). Key characteristics of the patients, including demographic and clinical data at the timepoint of BAL, are detailed in Table 1. The mean age was 57.5 ± 15.1 years, with females comprising 73% of the cohort. Disease duration for interstitial lung disease averaged 1.9 ± 2.9 years, disease duration for SARD averaged 6.0 ± 6.6 years and over half of the patients (51.7%) were non-smokers. NSIP emerged as the most frequent pattern on HRCT, observed in 58.4% of cases, while UIP appeared in 15.7%, and organizing pneumonia (OP) in 6.7%. The mean extent of lung involvement on HRCT measured 27.4 ± 18.4%. Regarding PFT, the average predicted FVC was 74.2 ± 21.7%, with a mean DLCO SB of 49.5 ± 18.2%. At the time of BAL, immunosuppressive treatment was used by 84.3% of the patients, including 32.6% on glucocorticoids and 26.7% on mycophenolate mofetil. Rituximab was administered to 10.1% of the cohort, primarily among those with SSc-ILD. Concomitant antifibrotic agents (i.e., nintedanib) were prescribed to 7.9% of the patients. The time points of baseline and follow-up assessments are depicted in Supplementary Figure S1.

### 3.2. BALF Findings

Lymphocytosis (defined as >15% lymphocytes) was present in 37.1% of patients, with the highest frequency in IIM-ILD (53.3%) and SS-ILD (50.0%). Neutrophilia (>3%) was observed in 37.1% of the cohort, most commonly in RA (53.3%) and MCTD (43.5%). Eosinophilia (>1%) was noted in 28.1% of patients. The CD4/CD8 ratio was variable across diagnoses, with a mean of 1.3 ± 1.3. Patients with IPAF showed the highest CD4/CD8 ratio (3.4 ± 2.4), while those with IIM-ILD exhibited the lowest (0.7 ± 0.6). Supplementary Table S1 provides detailed information regarding cellular differential distribution and immunophenotypic outcomes of BALF across the cohort.

### 3.3. Association Between BAL Lymphocyte Proportion and Disease Progression (Univariate Analysis)

We identified 30 patients (33.7%) as progressors and 59 (66.3%) as non-progressors. Among the 30 patients classified as progressors, four (13.3%) died due to ILD-related complications, one (3.3%) underwent lung transplantation, and ten (33.3%) were classified as progressors based on treatment intensification during the observation period. A detailed distribution of patients fulfilling specific subcriteria for pulmonary disease progression is provided in Supplementary Table S2. In the univariate analysis, the proportion of lymphocytes in BAL was significantly higher in patients with progressive SARD-ILD compared to non-progressive cases (progressors: 31.6 ± 24.8% vs. non-progressors: 14.3 ± 16.5%; *p* < 0.001). When examining other cellular types, no significant differences were observed in neutrophil or eosinophil proportions between the two groups. These findings, along with other clinical and cellular profile details, are presented in Supplementary Table S3. Similarly, when using the INBUILD criteria alone to define disease progression, the association between elevated BAL lymphocyte proportions and disease progression remained significant (progressors: 28.4 ± 28.0% vs. non-progressors: 18.2 ± 18.9%; *p* = 0.04). Further detailed results of univariate analysis are available in Supplementary Table S4.

### 3.4. Predicting Disease Progression with BAL Lymphocyte Proportion (Multivariable Logistic Regression Analysis)

Across all models, the BAL lymphocyte proportion remained significantly associated with disease progression (Supplementary Table S5). In the baseline model, the inclusion of lymphocyte proportion resulted in a pseudo-R^2^ of 29.3%, compared to 10.0% in the model without lymphocyte proportion (AIC: 102.9 vs. 115.2). The corresponding odds ratio (OR) for lymphocyte proportion in the baseline model was 1.05 (95% CI: 1.02–1.07, *p* < 0.01), indicating that each percentage increase in BAL lymphocyte proportion was associated with a 5% higher likelihood of disease progression (Supplementary Table S5). The model with all variables demonstrated the highest predictive power (Table 2). Incorporating BAL lymphocyte proportion achieved a pseudo-R^2^ of 58.7% and an AIC of 100.4, signifying a stronger model’s predictive performance compared to the model without lymphocytes, which had a pseudo-R^2^ of 39.8% and an AIC of 116.9 (Supplementary Table S5). BAL lymphocyte proportion consistently showed a significant association with disease progression, with incremental improvements in model fit and predictive power as reflected by higher pseudo-R^2^ values and lower AIC scores. Given the observational design and lack of external validation, these findings should be considered exploratory. Detailed results and comparisons between models can be found in Table 3. Clinical data stratified by BAL lymphocyte proportion are provided in Supplementary Table S6. Parameters from the differential blood count, acute-phase reactants (CRP) and other leukocyte subsets from the BAL differential cell count did not yield significant results (Supplementary Tables S7–S9).

To further address the potential impact of incorporation bias related to treatment intensification, additional sensitivity analyses were performed using a revised objective progression endpoint consisting exclusively of original INBUILD progression criteria, ILD-related mortality, and lung transplantation. In these analyses, BAL lymphocyte proportion remained independently associated with objective disease progression in multivariable analysis (OR 1.04, 95% CI 1.00–1.10, *p* = 0.03) (Supplementary Table S10, additional ROC and Kaplan–Meier analyses for this endpoint in Supplementary Figures S5–S7). Additional subgroup analyses according to BAL indication demonstrated that the association between BAL lymphocyte proportion and disease progression remained present in patients undergoing BAL during diagnostic evaluation of newly diagnosed ILD as well as in patients undergoing BAL for pre-existing/progressive ILD. Due to the limited number of progression events within the individual BAL indication subgroups, the full multivariable main model could not be reliably applied because of model instability and convergence problems. Therefore, subgroup analyses for the composite progression endpoint were performed using our foundational model adjusted for age, FVC, and HRCT extent. Within this model, no evidence of a significant interaction between BAL indication and BAL lymphocyte proportion was observed (interaction *p* = 0.55; Supplementary Table S11). Also, sensitivity analyses using Firth’s penalized logistic regression demonstrated consistent associations between BAL lymphocyte proportion and all three progression endpoints, including original INBUILD progression, the endpoint including INBUILD, lung transplantation and ILD-related mortality, and the composite progression endpoint (Supplementary Table S12).

Overall, follow-up HRCT imaging was available in 69/89 patients (77.5%), whereas 20/89 patients (22.5%) did not undergo repeat HRCT during follow-up. To further address the potential impact of differential imaging follow-up, we repeated the full multivariable analysis for the composite progression endpoint exclusively in patients with available follow-up HRCT imaging. In this restricted complete-imaging cohort, BAL lymphocyte proportion remained independently associated with disease progression (OR 1.08, 95% CI 1.03–1.14, *p* = 0.002).

### 3.5. Progression-Free Survival and Time-to-Disease-Progression

**Figure 1:**
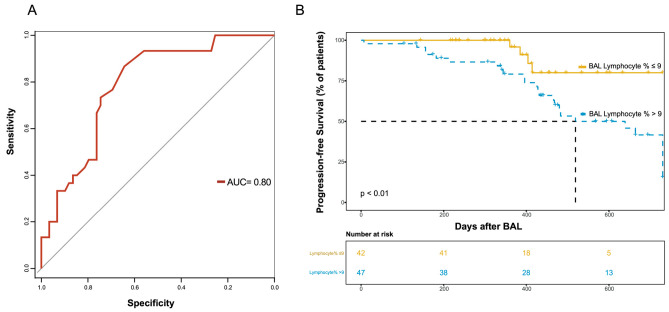
ROC curve and Kaplan–Meier analysis for BAL lymphocyte proportion in predicting disease progression. (**A**) The ROC curve illustrates the performance of BAL lymphocyte proportion in discriminating between progressive and non-progressive SARD-ILD, with an AUC of 0.80 (95% CI: 0.70–0.89). The optimal cutoff identified was 9.5%, based on the Youden Index. (**B**) The Kaplan–Meier analysis stratifies patients by BAL lymphocyte proportion (>9% vs. ≤9%) and demonstrates a significant difference in progression-free survival (*p* < 0.01). SARD-ILD patients with a lymphocyte proportion > 9% had a median progression-free survival of 519 days, while those with ≤9% did not reach the median during the observation period. Abbreviations: AUC, area under the curve; CI, confidence interval; BAL, bronchoalveolar lavage; SARD-ILD, systemic autoimmune rheumatic disease-associated interstitial lung disease; ROC, receiver operating characteristic; %, proportion.

To evaluate the predictive value of BAL lymphocyte proportion for progression-free survival (PFS), we first performed a receiver operating characteristic (ROC) analysis. The analysis yielded an area under the curve (AUC) of 0.80 (95% confidence interval: 0.70–0.89) (Figure 1A) for discriminating progressive versus non-progressive ILD. To identify a robust and clinically meaningful threshold, two complementary statistical approaches were applied. ROC analysis using Youden Index optimization identified an optimal BAL lymphocyte proportion cutoff of approximately 9.5%, whereas a standardized Wilcoxon-based binary threshold analysis yielded a highly similar optimal cutoff of 9% (Supplementary Figure S2). Given the close agreement between both methods, all subsequent binary analyses were standardized using a BAL lymphocyte cutoff of >9%. This cutoff demonstrated a sensitivity of 86.7%, specificity of 64.4%, positive predictive value of 55.3%, and negative predictive value of 90.5% for predicting disease progression, corresponding to 21 false-positive and 4 false-negative classifications. Positive and negative likelihood ratios were 2.44 and 0.21. In addition to the single-parameter model as shown in Figure 1A, we constructed a multivariable ROC analysis based on clinical parameters. The addition of BAL lymphocyte proportion in the final step (Model 4) was associated with the highest predictive accuracy for identifying patients at risk for progression (AUC = 0.89; 95% CI: 0.83–0.96) (Supplementary Figure S3).

Subsequent Kaplan–Meier analysis based on this BAL lymphocyte proportion threshold (>9% vs. ≤9%) revealed a significant difference in time to progression (Figure 1B). SARD-ILD patients with BAL lymphocyte proportion > 9% had a median progression-free survival of 519 days after BAL performance, while those with ≤9% had not reached the median during the observation period (*p* < 0.01). Also, in the multivariable analysis, a BAL lymphocyte proportion > 9% was associated with disease progression with an odds ratio of 13.14 (95% CI: 4.20–51.98, *p* < 0.01) (Supplementary Table S13). The association persisted when applying the ATS guideline BAL lymphocyte proportion threshold of >15% (Supplementary Table S14, Supplementary Figure S4). Additional sensitivity analyses using Firth-penalized Cox proportional hazards regression for the composite progression endpoint with the same covariate demonstrated consistent results, with BAL lymphocyte proportion remaining independently associated with shorter progression-free survival (HR 1.03, 95% CI 1.00–1.05, *p* = 0.03).

## 4. Discussion

Analyzing cells in the alveolar compartment through BAL may provide clinically relevant information on disease activity and progression in patients with SARD-ILD. The purpose of this study was to assess the value of BAL to identify patients at risk for progressive disease. The heterogenous course of SARD-ILD underlines the need for predictors of disease progression in order to initiate timely treatment or referral for lung transplantation in patients at risk.

In this cohort, 34% of patients developed progressive disease using a composite progression definition consisting of the INBUILD criteria, disease-specific mortality, lung transplantation or escalation of immunosuppressive therapy due to respiratory worsening [9]. This proportion is consistent with prior large observational studies reporting progression rates of 29–39% in SARD-ILD cohorts when defining progression using INBUILD enrolment criteria [36,37]. In line with previous studies, patients with progressive disease more frequently presented with IIM and less frequently with MCTD, reflecting known differences in disease behaviour across SARD subtypes and supporting the external validity of our cohort [38,39]. Although BAL lymphocyte proportion remained independently associated with disease progression after adjustment for the underlying SARD diagnosis, the limited number of patients within individual disease subgroups precluded robust disease-specific analyses. Moreover, the biological and prognostic significance of BAL lymphocytosis may differ across individual SARD-ILD entities and HRCT phenotypes, therefore likely reflecting heterogeneous inflammatory and fibrotic mechanisms between autoimmune disease subtypes. Therefore, our findings should not be interpreted as implying identical prognostic relevance of BAL lymphocytosis across all SARD-ILD subgroups. Larger multicentre studies are needed to confirm these findings across specific SARD-ILD subtypes.

Baseline HRCT patterns differed between progressors and non-progressors. 46.7% of patients with progressive disease presented with NSIP compared to 64.4% with non-progressive disease. Furthermore, 13.3% of patients with progressive disease presented with HRCT changes compatible with organizing pneumonia compared to a mere 3.4% in non-progressors. No significant changes in the extent of ILD on imaging, percent of FVC predicted, percent of DLCO predicted, smoking status and sex were found in-between groups.

The key novel finding of our study is that BAL fluid (BALF) lymphocyte proportion was more than twofold higher in patients who developed progressive disease and remained independently associated with progression after adjusting for potential confounders. Patients with a BALF lymphocyte count above 9 percent demonstrated a 13-fold increased risk of developing progressive interstitial lung disease. As this threshold was derived within the present cohort and has not been externally validated, its prognostic performance should be considered exploratory and hypothesis-generating. Notably, established markers of progression such as FVC, DLCO, and HRCT extent of disease were not independently associated with progression in our multivariable models, with the exception of age. This may be explained by the heterogeneity of our cohort in terms of SARD diagnoses, disease duration and timing of ILD manifestation (new-onset vs. progressive disease).

Data on the prognostic value of BALF differential cell counts in SARD-ILD are scarce and predominantly derived from studies in systemic sclerosis-associated ILD. Hoffmann et al. identified a lymphocytic BAL cellular pattern as the most prevalent finding, occurring in 41.9% of 74 patients with inflammatory rheumatic disease–associated interstitial lung disease, compared with 37.1% in our cohort [40]. However, the authors did not evaluate its association with disease progression. Contrary to our findings, Goh et al. found no association between BALF lymphocytosis and disease progression in a cohort of 134 patients with systemic sclerosis–associated interstitial lung disease [41]. However, the presence of BAL neutrophils > 4% was associated with increased mortality. This discrepancy with our findings may reflect differences in progression definitions focused on functional decline or death, without incorporating radiographic progression or treatment escalation.

Beyond SSc-ILD, most prior BAL studies in interstitial lung disease have concentrated on idiopathic interstitial pneumonias rather than autoimmune disease. In idiopathic pulmonary fibrosis, identified increased BALF neutrophil counts have repeatedly been associated with disease progression and mortality [42,43]. Barnett et al. showed that BAL lymphocytosis ≥ 25% was associated with a lower likelihood of disease progression in a cohort of patients with IPF, fibrotic HP, unclassifiable ILD and idiopathic NSIP [44]. Our findings suggest that BAL cellular profiles may reflect disease-specific immunopathological mechanisms that differ between SARD-ILD and idiopathic interstitial lung diseases.

Our study provides several distinctive and clinically relevant contributions. Firstly, to our knowledge this is the largest study, specifically designed to evaluate BAL differential cell counts as potential predictors of disease progression in a well-characterized SARD-ILD cohort with longitudinal follow-up. Unlike prior work focused on SSc-ILD alone, our cohort comprised several SARD-ILD subtypes, providing data from a heterogeneous patient population. However, the findings should not be interpreted as establishing equivalent associations across all individual SARD-ILD subgroups. Secondly, we employed a robust composite outcome for defining disease progression by implementing the broadly validated and evidence-based INBUILD criteria, disease-specific mortality and immunosuppressive therapy extension based on the clinician’s assessment.

Thirdly, our findings suggest that BAL lymphocytosis identifies a subgroup of SARD-ILD patients in whom alveolar inflammation persists despite comparable baseline lung function and radiographic extent of disease. This may reflect ongoing alveolar inflammation that eventually translates into fibrosis progression, functional decline, or treatment escalation, supporting the utility of BAL as a complementary tool to aid in the earlier identification of patients at risk for disease progression. However, given the observational design of the study, these findings should be interpreted as associations and do not establish a causal relationship between BAL lymphocytosis and subsequent disease progression.

This study is subject to several limitations. The single-centre study design may lead to potential bias. As patients received bronchoscopy due to new onset or clinically suspected progressive ILD, this study likely suffered from selection bias and confounding by indication. Although we adjusted for disease duration in our models, this variable may not fully account for clinical factors present at the time of BAL. Elevated BAL lymphocyte proportions may reflect several biological processes beyond persistent alveolar inflammation related to the underlying autoimmune disease. BAL lymphocytosis can also be observed in the setting of infection, organizing pneumonia, drug-induced lung injury, and other interstitial lung disease phenotypes. Although bronchoalveolar lavage was performed as part of the diagnostic evaluation and all cases were reviewed in a multidisciplinary discussion, BAL lymphocytosis is not disease-specific and may represent a range of overlapping pathological processes. Nonetheless, our findings suggest that, irrespective of the underlying mechanism, increased lymphocyte proportions in the alveolar compartment may identify patients with SARD-ILD at increased risk of subsequent disease progression.

Also, follow-up HRCT data was not available for every patient as follow-up HRCT was only performed when clinically indicated. Although sensitivity analyses restricted to patients with available follow-up HRCT imaging showed consistent results, the potential for residual detection bias due to the retrospective study design cannot be excluded. Changes in respiratory symptoms, imaging findings, or the initiation of immunosuppressive therapy could have influenced the decision to perform BAL and the lymphocyte proportions, respectively. Furthermore, treatment escalation may have been influenced by BAL findings themselves, potentially introducing incorporation bias when therapeutic intensification was included within the composite progression endpoint. Therefore, residual confounding remains a limitation and may have affected the strength of our findings. The retrospective single-centre design and the relatively limited number of progression events restricted statistical power for adequately powered subgroup analyses across individual SARD-ILD diagnoses and HRCT patterns. Therefore, subgroup-specific findings should be interpreted with caution.

## 5. Conclusions

This study found that the BALF lymphocyte proportion was independently associated with disease progression according to a composite progression definition. These findings contribute novel insight to an area with limited prior evidence, challenging the prevailing notion that BAL cellular analysis lacks prognostic relevance in autoimmune-related ILD. Further multicentre trials in different SARD-ILD subtypes are needed to validate these findings and investigate the underlying mechanisms linking immune cells in the alveolar compartment and progression of interstitial lung disease, potentially exploring the role of specific lymphocyte subsets. Bronchoscopy with BAL may complement existing tools to improve risk stratification and support more personalized clinical decision-making.

## Figures and Tables

**Table 1:** Demographic and clinical features of patients with SARD-ILD stratified according to diagnosis at baseline (timepoint of BAL).

Variable	All (*n* = 89)	Myositis (*n* = 15)	Systemic Sclerosis (*n* = 21)	Rheumatoid Arthritis (*n* = 15)	Mixed Connective Tissue Disease (*n* = 23)	Sjögren’s Syndrome (*n* = 8)	Other (*n* = 7)
Age (y, mean ± SD)	57.5 ± 15.1	49.2 ± 16.6	50.6 ± 12.9	69.7 ± 10.4	56.6 ± 14.0	62.6 ± 15.1	66.4 ± 9.0
Female Sex (*n*, %)	65 (73.0)	10 (66.7)	17 (81.0)	8 (53.3)	20 (86.9)	6 (75.0)	4 (57.1)
Disease duration of ILD (y, mean ± SD)	1.9 ± 2.9	2.9 ± 3.4	1.4 ± 2.6	1.3 ± 2.4	2.9 ± 3.3	0.9 ± 2.1	0.3 ± 0.8
Disease duration of SARD (y, mean ± SD)	6.0 ± 6.6	3.3 ± 4.6	5.4 ± 5.1	9.0 ± 8.8	7.3 ± 7.1	5.1 ± 6.1	4.4 ± 7.1
Smokers (*n*, %)							
Never	46 (51.7)	8 (53.3)	12 (57.1)	3 (20.0)	16 (69.6)	6 (75.0)	1 (14.3)
Former	37 (41.6)	7 (46.7)	9 (42.9)	11 (73.3)	3 (13.0)	2 (25.0)	5 (71.4)
Current	6 (6.7)	0 (0.0)	0 (0.0)	1 (6.7)	4 (17.4)	0 (0.0)	1 (14.3)
FVC (% predicted, mean ± SD)	74.2 ± 21.7	64.5 ± 22.3	76.4 ± 21.6	79.2 ± 18.4	71.0 ± 21.5	87.1 ± 28.4	72.7 ± 14.3
DLCO SB (% predicted, mean ± SD)	49.5 ± 18.2	47.1 ± 19.0	54.7 ± 18.6	51.7 ± 16.2	46.2 ± 18.3	52.1 ± 20.0	39.8 ±17.3
DLCO/VA (% predicted, mean ± SD)	69.9 ± 17.0	73.0 ± 22.5	72.4 ± 15.4	74.7 ± 21.0	66.0 ± 12.2	66.9 ± 14.8	59.6 ± 12.4
HRCT pattern (*n*, %)							
NSIP	52 (58.4)	10 (66.7)	17 (81.0)	5 (33.3)	13 (56.5)	4 (50.0)	3 (42.9)
UIP	14 (15.7)	0 (0.0)	3 (14.3)	4 (26.7)	6 (26.1)	0 (0.0)	1 (14.3)
OP	6 (6.7)	3 (30.0)	1 (4.8)	0 (0.0)	2 (8.7)	0 (0.0)	0 (0.0)
Unclassified	10 (11.2)	2 (13.3)	0 (0.0)	6 (40.0)	0 (0.0)	0 (0.0)	2 (28.6)
LIP	4 (4.5)	0 (0.0)	0 (0.0)	0 (0.0)	0 (0.0)	4 (50.0)	0 (0.0)
Other	3 (3.4)	0 (0.0)	0 (0.0)	0 (0.0)	2 (8.7)	0 (0.0)	1 (14.3)
Total disease extent on HRCT (%, mean ± SD)	27.4 ± 18.4	28.3 ± 16.9	20.0 ± 14.7	24.3 ± 18.4	33.5 ± 19.9	20.8 ± 8.6	39.3 ± 24.4
Immunosuppressive treatment (*n*, %)	75 (84.3)	15 (100.0)	16 (76.2)	15 (100.0)	20 (86.9)	7 (87.5)	2 (28.6)
Glucocorticoids	29 (32.6)	9 (60.0)	3 (14.3)	4 (26.7)	9 (39.1)	3 (37.5)	1 (14.3)
Rituximab	9 (10.1)	1 (6.7)	4 (19.0)	0 (0.0)	4 (17.4)	0 (0.0)	0 (0.0)
Mycophenolate mofetil	24 (26.7)	6 (40.0)	9 (42.9)	0 (0.0)	5 (21.7)	3 (37.5)	1 (14.3)
Methotrexate	11 (12.4)	1 (6.7)	4 (19.0)	5 (33.3)	1 (4.3)	0 (0.0)	0 (0.0)
Leflunomide	2 (2.2)	0 (0.0)	0 (0.0)	2 (13.3)	0 (0.0)	0 (0.0)	0 (0.0)
Azathioprine	2 (2.2)	0 (0.0)	0 (0.0)	1 (6.7)	1 (4.3)	0 (0.0)	0 (0.0)
Tocilizumab	5 (5.6)	0 (0.0)	1 (4.8)	1 (6.7)	3 (13.0)	0 (0.0)	0 (0.0)
Antifibrotic treatment (*n*, %)	7 (7.9)	2 (13.3)	1 (4.8)	1 (6.7)	3 (13.0)	0 (0.0)	0 (0.0)
Peripheral blood parameters (mean ± SD)							
Leukocytes (absolute in G/L)	7.9 ± 2.9	8.2 ± 3.0	7.5 ± 2.5	9.8 ± 3.3	7.1 ± 2.5	9.0 ± 2.6	6.1 ± 1.6
Lymphocytes (absolute in G/L)	1.5 ± 0.7	1.3 ± 0.6	1.7 ± 0.7	1.5 ± 0.7	1.2 ± 0.6	1.3 ± 0.7	1.7 ± 0.7
Lymphocytes (relative in %)	19.8 ± 9.0	18.0 ± 7.7	23.2 ± 9.3	17.6 ± 8.5	16.8 ± 6.7	16.4 ± 8.3	29.8 ± 10.3
Monocytes (absolute in G/L)	0.6 ± 0.3	0.5 ± 0.2	0.6 ± 0.3	0.6 ± 0.2	0.6 ± 0.3	0.7 ± 0.3	0.5 ± 0.2
Monocytes (relative in %)	7.7 ± 3.0	7.6 ± 3.2	7.5 ± 2.5	7.2 ± 2.9	8.4 ± 3.6	7.4 ± 2.8	8.0 ± 3.1
CRP in mg/dL	1.0 ± 1.6	1.1 ± 1.2	1.2 ± 2.4	1.3 ± 1.5	0.9 ± 1.4	0.7 ± 0.5	0.3 ± 0.2

Abbreviations: *n*, number; y, years; SD, standard deviation; SARD, systemic autoimmune rheumatic disease; ILD, interstitial lung disease; FVC (% predicted), forced vital capacity; DLCO (% predicted), diffusion capacity of the lungs for carbon monoxide; SB, single breath; VA, alveolar volume; HRCT, high-resolution computed tomography; NSIP, nonspecific interstitial pneumonia; UIP, usual interstitial pneumonia; OP, organizing pneumonia; LIP, lymphocytic interstitial pneumonia; CRP, C-reactive protein; G/L, giga per litre; mg/dL, milligram per deciliter.

**Table 2:** Logistic regression model for predicting disease progression with lymphocyte proportion in BAL.

Variable		OR	95% CI Lower	95% CI Upper	*p*-Value
Age (years)		1.11	1.03	1.21	0.01
FVC (% predicted)		0.98	0.93	1.02	0.26
Total disease extent on HRCT (%)		0.98	0.93	1.04	0.51
Lymphocyte proportion (%)		1.08	1.04	1.14	<0.01
Sex (male)		0.79	0.16	3.81	0.77
Disease duration (years)		1.12	0.87	1.45	0.39
Treatment (yes)					
	Rituximab	0.45	0.02	6.03	0.57
	Mycofenolate mofetil	0.93	0.17	5.30	0.94
	Glucocorticoids	0.32	0.04	2.14	0.26
	Antifibrotic	0.80	0.05	11.73	0.87
DLCO (% predicted)		0.98	0.92	1.03	0.46
SARD-Diagnosis (Reference: Myositis)					
	Systemic Sclerosis	3.55	0.41	37.46	0.26
	Rheumatoid Arthritis	0.23	0.02	2.83	0.26
	Mixed Connective Tissue Disease	0.21	0.02	1.97	0.19
	Sjögren’s Syndrome	1.16	0.07	23.57	0.92
	Other	1.09	0.06	21.69	0.95
Pseudo-R^2^ = 58.7%					
AIC = 100.4					

This table presents the results of logistic regression analysis for predicting the outcome of disease progression with the inclusion of lymphocyte proportion in BAL. The baseline model included age, FVC (% predicted), extent of disease on HRCT, and lymphocyte proportion in BAL. This model further incorporated additional variables such as sex, disease duration, specific treatments, DLCO (% predicted), and SARD diagnoses (without inclusion of the HRCT pattern, as this may introduce collinearity with the SARD diagnosis). Model performance is evaluated using pseudo-R^2^ and the Akaike Information Criterion (AIC). *p*-values < 0.05 are considered statistically significant. Abbreviations: SARD, systemic autoimmune rheumatic disease; FVC (% predicted), forced vital capacity; HRCT, high-resolution computed tomography; DLCO (% predicted), diffusing capacity of the lungs for carbon monoxide; OR, odds ratio; CI, confidence interval; AIC, Akaike Information Criterion; Pseudo-R^2^ (model fit).

**Table 3:** Comparison of model performance with and without BAL lymphocyte proportion for predicting disease progression in SARD-ILD.

Model		Baseline	+ Sex	+ Disease Duration	+ Treatment	+ DLCO	+ SARD Diagnosis	+ HRCT Pattern	All and SARD-Diagnosis
With Lymphocytes %	R^2^	29.3	30.0	29.3	30.5	44.9	45.1	33.3	58.7
	AIC	102.9	104.3	104.8	109.8	92.3	99.1	109.5	100.4
Without Lymphocytes %	R^2^	10.0	11.0	10.7	13.8	24.9	29.3	23.8	39.8
	AIC	115.2	116.3	116.7	120.5	106.9	110.8	115.2	116.9

This table presents the results of multivariable logistic regression models assessing the predictive power of various clinical, pulmonary, radiographic, and therapeutic factors for disease progression in SARD-ILD. Starting from the baseline model, which included age, FVC, and extent of disease on HRCT, additional variables were added sequentially, including sex, disease duration, treatment regimen, DLCO, SARD diagnosis, and HRCT pattern. The table compares models with and without the inclusion of BAL lymphocyte proportion, reporting pseudo-R^2^ and Akaike Information Criterion (AIC) values for each model. The inclusion of BAL lymphocyte proportion consistently improved the predictive strength of the models, with the final model incorporating all variables and SARD diagnosis showing the highest predictive power (without HRCT pattern, as it may be collinear with the SARD diagnosis). Abbreviations: SARD, systemic autoimmune rheumatic disease; ILD, interstitial lung disease; %, proportion; FVC (% predicted), forced vital capacity; DLCO (% predicted), diffusing capacity of the lungs for carbon monoxide; HRCT, high-resolution computed tomography; AIC, Akaike Information Criterion; Pseudo-R^2^ (model fit).

## Data Availability

All study-relevant data are either presented within this article or available as Online Supplementary Materials. De-identified patient data will be made available upon reasonable request to the corresponding author (marco.idzko@meduniwien.ac.at). Requests must include a brief proposal outlining the purpose and rationale for data access and will be considered on an individual basis. Data will be shared after approval of the proposal and completion of a signed data access agreement.

## Supplementary Materials

The following supporting information can be downloaded at: https://www.mdpi.com/article/10.3390/jcm15124834/s1, Figure S1: Timeline of assessments; Figure S2: Standardized Wilcoxon statistic for identification of the optimal cutoff of BAL lymphocyte; Figure S3: Receiver operating characteristic (ROC) models comparing prediction of disease progression using clinical parameters alone versus with BAL lymphocyte proportion; Figure S4: Kaplan–Meier analysis for BAL lymphocyte proportion in predicting disease progression; Figure S5: Receiver operating characteristic (ROC) models comparing prediction of disease progression using clinical parameters alone vs. with BAL lymphocyte proportion; Figure S6: Kaplan–Meier analysis for progression-free survival according to bronchoalveolar lavage (BAL) lymphocyte proportion using a cut-off of 9; Figure S7: Kaplan–Meier analysis for progression-free survival according to bronchoalveolar lavage (BAL) lymphocyte proportion using a cut-off of 15%; Table S1: Bronchoalveolar lavage fluid (BALF) findings across patients with SARD-ILD, stratified by SARD diagnosis; Table S2: Distribution of SARD-ILD patients classified as having disease progression according to the INBUILD criteria versus the composite endpoint; Table S3: Clinical and cellular characteristics of patients with progressive and non-progressive SARD-ILD; Table S4: Clinical and cellular characteristics of patients with progressive and non-progressive SARD-ILD, applying the original INBUILD criteria; Table S5: Multivariable logistic regression models for predicting disease progression in SARD-ILD; Table S6: Clinical and cellular characteristics of patients with SARD-ILD stratified by BAL lymphocyte proportion (≤9% vs. >9%); Table S7: Multivariable logistic regression analysis including eosinophil proportion in BAL for predicting disease progression in SARD-ILD; Table S8: Multivariable logistic regression analysis including neutrophil proportion in BAL for predicting disease progression in SARD-ILD; Table S9: Multivariable logistic regression analysis including peripheral blood parameters and BAL lymphocyte proportion for predicting disease progression in SARD-ILD; Table S10: Multivariable logistic regression analysis for the progression endpoint of INBUILD, ILD-related mortality, or lung transplantation, excluding treatment escalation; Table S11: Subgroup and interaction analyses evaluating the association between bronchoalveolar lavage (BAL) lymphocyte proportion and disease progression according to BAL indication at baseline; Table S12: Multivariable Firth penalized logistic regression analyses evaluating the association between bronchoalveolar lavage (BAL) lymphocyte proportion and disease progression across three different progression endpoints, including original INBUILD progression criteria, the endpoint consisting of INBUILD progression, ILD-related mortality or lung transplantation, and the composite progression endpoint including treatment escalation; Table S13: Multivariable logistic regression analysis including BAL lymphocyte proportion > 9% for predicting disease progression in SARD-ILD; Table S14: Multivariable logistic regression analysis including BAL lymphocyte proportion > 15% for predicting disease progression in SARD-ILD. References [35,45,46] are cited in the Supplementary Materials.

## Abbreviations

The following abbreviations are used in this manuscript:

AIC	Akaike Information Criterion
ATS	American Thoracic Society
AUC	Area under the curve
BAL	Bronchoalveolar lavage
BALF	Bronchoalveolar lavage fluid
CD	Cluster of differentiation
CI	Confidence interval
CRP	C-reactive protein
CT	Computed tomography
CTD	Connective tissue disease
DLCO	Diffusing capacity of the lungs for carbon monoxide
EMR	Electronic medical records
FVC	Forced vital capacity
GC	Glucocorticoids
HRCT	High-resolution computed tomography
IIM	Idiopathic inflammatory myositis
ILD	Interstitial lung disease
IPAF	Interstitial pneumonia with autoimmune features
IPF	Idiopathic pulmonary fibrosis
IQR	Interquartile range
LIP	Lymphocytic interstitial pneumonia
LTOT	Long-term oxygen therapy
MCTD	Mixed connective tissue disease
MDD	Multidisciplinary discussion
NSIP	Nonspecific interstitial pneumonia
NYHA	New York Heart Association
OP	Organizing pneumonia
OR	Odds ratio
PFS	Progression-free survival
PFT	Pulmonary function test
RA	Rheumatoid arthritis
RMD	Rheumatic and musculoskeletal disease
ROC	Receiver operating characteristic
SARD	Systemic autoimmune rheumatic disease
SARD-ILD	Systemic autoimmune rheumatic disease-associated interstitial lung disease
SD	Standard deviation
SLE	Systemic lupus erythematosus
SS	Sjögren’s syndrome
SSc	Systemic sclerosis
STROBE	Strengthening the Reporting of Observational Studies in Epidemiology
TBLC	Transbronchial lung cryobiopsy
UIP	Usual interstitial pneumonia
