# Supplementary material for: Prognostic Value of Bronchoalveolar Lavage in Systemic Autoimmune Rheumatic Diseases-Associated Interstitial Lung Disease
Source: J Clin Med. 2026 Jun 22;15(12):4834. doi: 10.3390/jcm15124834 (PMC13301357; doi:10.3390/jcm15124834)
Supplement: Supplementary file 1 [file jcm-15-04834-s001.zip › jcm-4308610-supplementary.pdf]

**Prognostic value of bronchoalveolar lavage in systemic autoimmune rheumatic diseases-associated interstitial lung disease**

**Supplementary Material**

## Supplementary Methods

### Bronchoalveolar lavage protocol

The bronchoscope was wedged in the segmental or subsegmental bronchus of the middle lobe or the lingula. Up to 300ml sterile saline was injected stepwise via a handheld syringe and then gradually withdrawn back into the syringe. The BALF was prepared and assessed for total cell count. In patients with ≥ 11% lymphocytes in BALF samples flow cytometry analysis was performed assessing CD4/CD8 ratio, proportions of CD3+ T cells, CD4+ helper T cells, CD8+ cytotoxic T cells, B cells (CD19+) and natural killer cells (CD57+). After counting and washing, Fc receptors were blocked for 30 minutes using Human TruStain FcX (BioLegend). Then a 20-minute FACS staining in phosphate-buffered saline containing 0.5% bovine serum albumin and 0.01% sodium azide was applied. Differential cell counts were analyzed using flow cytometry. Analysis was performed on a CytoFlex LS (Beckman Coulter) using Cellquest version 3.3 (BD Bioscineces) and FlowJo version 6.4.7 (Tree Star Inc.) software. Thresholds for BAL cell patterns were derived from the ATS clinical practice guideline, i.e. >15% for lymphocytes, >3% pathological or neutrophils and >1% for eosinophils [1]

### Analysis of HRCT and semiquantitative scoring of ILD extent.

HRCT chest scans were evaluated by two experienced radiologists in lung imaging. CT scans were assessed for the presence and overall extent of ILD-related abnormalities, utilizing a semi-quantitative scoring system based on the visual staging method introduced by Goh et al. [2]. This approach specifically considered variables such as the total extent of disease and the proportion of ground-glass opacities, defined as areas of increased lung opacity with preserved visibility of bronchial and vascular structures. In brief, the extent of lung involvement was estimated in five predetermined pulmonary sections: 1. origin of the great vessels; 2. main carina; 3. pulmonary venous confluence; 4. midway between the third and fifth sections, and 5.

just above the right hemidiaphragm, with increments of 5% used for each estimate. Furthermore, the CT scans were examined for signs of bronchial wall thickening, bronchiectasis, consolidation (characterized by a uniform increase in lung tissue density that obscures vascular and airway margins, sometimes with air bronchograms), reticular patterns (marked by the thickening of interlobular or intralobular septa), honeycombing (clusters of cystic airspaces with similar diameters), and emphysema, following the definitions provided by the Fleischner Society's glossary of thoracic imaging terms [3]. The evaluations by both radiologists were combined to produce a consensus on semi-quantitative HRCT scores, and the determination of the specific pattern or rather subtype of ILD for each patient with SARD.

## Supplementary Tables

**Table S1: Bronchoalveolar lavage fluid (BALF) findings across patients with SARD-ILD, stratified by SARD diagnosis.** The table includes parameters such as BALF recovery volume (ml), total cell count (cells/100 mL), and the differential distribution of neutrophils, lymphocytes, eosinophils, macrophages. Immunophenotypic data (e.g., CD4/CD8 ratio) were also evaluated. Abbreviations: BALF, bronchoalveolar lavage fluid; No./n, number; SD, standard deviation; ml, milliliters; CD, cluster of differentiation.

| Parameter | All (n = 89) | Myositis (n=15) | Systemic Sclerosis (n=21) | Rheumatoid Arthritis (n=15) | Mixed connective tissue disease (n=23) | Sjögren's syndrome (n=8) | Other (n=7) |
|---|---|---|---|---|---|---|---|
| **Volume of BALF recovery (ml, mean ± SD)** | 137.9 ± 42.0 | 144.7 ± 46.1 | 150.1 ± 41.5 | 125.1 ± 33.3 | 135.7 ± 45.5 | 138.9 ± 51.1 | 120.0 ± 23.0 |
| **BALF total cell count (cells/100mL, mean ± SD)** | 11.6 ± 11.5 | 12.1 ± 13.8 | 12.2 ± 9.1 | 6.0 ± 8.7 | 14.9 ± 14.7 | 13.4 ± 8.9 | 7.3 ± 4.0 |
| **Neutrophils (%, mean ± SD, range)** | 11.1 ± 18.0 | 6.3 ± 9.3 | 5.8 ± 8.9 | 19.2 ± 25.9 | 9.8 ± 20.3 | 18.6 ± 19.6 | 11.0 ± 11.5 |
| **Lymphocytes (%, mean ± SD, range)** | 20.2 ± 21.2 | 31.3 ± 26.4 | 14.6 ± 11.1 | 17.2 ± 18.7 | 16.7 ± 23.5 | 28.0 ± 25.5 | 22.0 ± 19.5 |
| **Eosinophils (%, mean ± SD, range)** | 3.1 ± 2.7 | 3.4 ± 1.5 | 1.8 ± 1.2 | 3.3 ± 2.8 | 2.8 ± 2.1 | 9.7 ± 2.5 | 1.3 ± 0.6 |
| **Presence of Neutrophilia (> 3%), n (%)** | 33 (37.1) | 3 (20.0) | 6 (28.6) | 8 (53.3) | 10 (43.5) | 3 (37.5) | 3 (42.9) |
| **Presence of Lymphocytosis (> 15%), n (%)** | 33 (37.1) | 8 (53.3) | 8 (38.1) | 4 (26.7) | 6 (26.1) | 4 (50.0) | 3 (42.9) |
| **Presence of Eosinophilia (> 1%), n (%)** | 25 (28.1) | 5 (33.3) | 3 (14.3) | 5 (33.3) | 8 (34.8) | 3 (37.5) | 1 (14.3) |
| **Presence of Macrophages (> 80%), n (%)** | 41 (46.1) | 6 (40.0) | 13 (61.9) | 5 (33.3) | 13 (56.5) | 1 (12.5) | 3 (42.9) |
| **CD4/CD8 (mean ± SD, n)** | 1.3 ± 1.3 | 0.7 ± 0.6 (8) | 0.9 ± 0.4 (11) | 1.3 ± 0.4 (2) | 1.2 ± 0.7 (6) | 1.6 ± 1.4 (3) | 3.4 ± 2.4 (5) |
| **CD3 (%, mean ± SD, n)** | 82.3 ± 21.8 | 69.2 ± 30.7 (8) | 84.1 ± 19.5 (11) | 91.0 ± 1.4 (2) | 86.8 ± 19.1 (6) | 77.8 ± 26.5 (3) | 93.0 ± 7.7 (5) |
| **CD4 (%, mean ± SD, n)** | 37.7 ± 19.7 | 22.2 ± 17.7 (8) | 37.2 ± 13.3 (11) | 42.5 ± 3.5 (2) | 38.2 ± 17.2 (6) | 30.9 ± 11.1 (3) | 64.8 ± 20.6 (5) |
| **CD8 (%, mean ± SD, n)** | 39.8 ± 18.8 | 44.3 ± 23.9 (8) | 44.0 ± 13.7 (11) | 35.0 ± 7.1 (2) | 40.9 ± 20.9 (6) | 36.4 ± 30.1 (3) | 25.9 ± 13.1 (5) |
| **Presence of CD19 (≥ 4%), n (%)** | 3 (3.4) | 0 (0.0) | 2 (9.5) | 0 (0.0) | 1 (4.3) | 0 (0.0) | 0 (0.0) |
| **Presence of CD57 (≥ 2%), n (%)** | 12 (13.5) | 3 (20.0) | 4 (19.0) | 2 (13.3) | 2 (8.7) | 1 (12.5) | 0 (0.0) |

**Table S2. Composition of progression endpoint components in SARD-ILD patients classified as having disease progression according to the original INBUILD criteria, the progression endpoint including INBUILD progression, ILD-related mortality or lung transplantation, and the composite progression endpoint.** The composite definition additionally included treatment intensification. Categories are not mutually exclusive, as some patients fulfilled more than one progression subcriterion. Percentages are given relative to the total number of progressors per definition. Abbreviations: SARD-ILD, systemic autoimmune rheumatic disease-associated interstitial lung disease; N/A, not applicable.

| Criterion | Progress defined by INBUILD (n=17;≙100%) | Progress defined by INBUILD + death/LTX (n = 20; 100%) | Progress defined by Composite Endpoint (n=30; ≙100%) |
|---|---|---|---|
| **INBUILD** | 17 (100%) | 17 (85.0%) | 17 (56.7%) |
| **ILD-related mortality** | N/A | 4 (20.0%) | 4 (13.3%) |
| **Lung transplantation** | N/A | 1 (5.0%) | 1 (3.3%) |
| **Treatment intensification** | N/A | N/A | 10 (33.3%) |

**Table S3: Clinical and cellular characteristics of patients with progressive and non-progressive SARD-ILD.** This table presents a detailed comparison of demographic and clinical data including HRCT pattern, extent of pulmonary disease, pulmonary function test results, and bronchoalveolar lavage fluid (BALF) cellular profiles, such as lymphocyte, neutrophil, and eosinophil proportions, between progressive and non-progressive cases. Based on criteria for disease progression, over the 2-year observation period, 30 patients (33.7%) were identified as progressors and 59 (66.3%) were classified as non-progressors. Data are presented as mean ± standard deviation (SD) for continuous variables and as numbers (percentages) for categorical variables. Abbreviations: No./n, number; SARD-ILD, systemic autoimmune rheumatic disease-associated interstitial lung disease; BALF, bronchoalveolar lavage fluid; FVC (% predicted), forced vital capacity; DLCO (% predicted), diffusing capacity of the lungs for carbon monoxide; SD, standard deviation; HRCT, high-resolution computed tomography; NSIP, nonspecific interstitial pneumonia; UIP, usual interstitial pneumonia; OP, organizing pneumonia; LIP, lymphocytic interstitial pneumonia. BAL, bronchoalveolar lavage; CRP, C-reactive protein; G/L, giga per liter; mg/dl, milligram per deciliter. P-values <0.05 were considered statistically significant.

| **Variable** | **Progressive** | **Non-Progressive** | **p-value** |
|---|---|---|---|
| **No. of patients n (%)** | 30 (33.7) | 59 (66.3) | |
| **Male sex n (%)** | 10 (33.3) | 14 (23.7) | 0.33 |
| **Age (y, mean ± SD)** | 60.6 ± 14.3 | 55.9 ± 15.4 | 0.15 |
| **Disease duration (y, mean ± SD)** | 1.43 ± 2.76 | 2.12 ± 2.93 | 0.10 |
| **Ever-smoker n (%)** | 13 (43.3) | 30 (50.8) | 0.66 |
| **Immunosuppressive treatment n (%)** | 24 (80.0) | 51 (86.4) | 0.63 |
| **Antifibrotic treatment n (%)** | 2 (6.7) | 5 (8.5) | 1.00 |
| **Rituximab n (%)** | 2 (6.7) | 7 (11.9) | 0.71 |
| **Mycophenolate mofetil n (%)** | 8 (26.7) | 16 (27.1) | 1.00 |
| **Methotrexate n (%)** | 4 (13.3) | 7 (11.9) | 1.00 |
| **Leflunomide n (%)** | 0 (0.0) | 2 (3.4) | 0.55 |
| **Azathioprine n (%)** | 0 (0.0) | 2 (3.4) | 0.55 |
| **Tocilizumab n (%)** | 2 (6.7) | 3 (5.1) | 1.00 |
| **Glucocorticoids** | 12 (40.0) | 17 (28.8) | 0.41 |
| **FVC (% predicted, mean ± SD)** | 69.4 ± 19.4 | 76.6 ± 22.6 | 0.19 |
| **DLCO SB (% predicted, mean ± SD** | 44.4 ± 14.9 | 52 ± 19.3 | 0.10 |
| **DLCO/VA (% predicted, mean ± SD** | 67.9 ± 17.4 | 70.8 ± 16.9 | 0.51 |
| **SARD Diagnosis** | | | 0.04 |
| Myositis | 7 (23.3) | 8 (13.6) | 0.24 |
| Systemic Sclerosis | 8 (26.7) | 13 (22.0) | 0.63 |
| Rheumatoid Arthritis | 5 (16.7) | 10 (16.9) | 0.97 |
| Mixed connective tissue disease | 2 (6.7) | 21 (35.6) | 0.01 |
| Sjögren's syndrome | 4 (13.3) | 4 (6.8) | 0.31 |
| Others (IPAF, other) | 4 (13.3) | 3 (5.1) | 0.17 |
| **HRCT pattern n (%)** | | | 0.12 |
| NSIP | 14 (46.7) | 38 (64.4) | 0.11 |
| UIP | 5 (16.7) | 9 (15.2) | 0.86 |
| OP | 4 (13.3) | 2 (3.4) | 0.17 |
| LIP | 1 (3.3) | 3 (5.1) | 1.00 |
| Unclassified | 6 (20.0) | 4 (6.8) | 0.06 |
| Other | 0 (0.0) | 3 (5.1) | 0.55 |
| **Extent of disease on HRCT (%,mean ± SD)** | 28.5 ± 16.6 | 26.8 ± 19.4 | 0.42 |
| **BALF total cell count (cells/100ml, median, range)** | 12.6 ± 12.6 | 11.0 ± 11.0 | 0.58 |
| **BAL Neutrophils (%, mean ± SD, range)** | 8.5 ± 10.0 | 12.6 ± 21.1 | 0.99 |
| **BAL Lymphocytes (%, mean ± SD, range)** | 31.6 ± 24.8 | 14.3 ± 16.5 | **<0.001** |
| **BAL Eosinophils (%, mean ± SD, range)** | 3.2 ± 3.6 | 3.1 ± 2.3 | 0.65 |

| **Peripheral blood parameters (mean ± SD)** | | | |
|---|---|---|---|
| Leukocytes (absolute in G/L) | 8.0 ± 3.1 | 7.9 ± 2.8 | 0.72 |
| Lymphocytes (absolute in G/L) | 1.4 ± 0.6 | 1.5 ±0.7 | 0.34 |
| Lymphocytes (relative in %) | 18.3 ± 9.4 | 20.6 ± 8.8 | 0.12 |
| Monocytes (absolute in G/L) | 0.5 ± 0.2 | 0.6 ± 0.3 | 0.19 |
| Monocytes (relative in %) | 6.9 ± 2.7 | 8.2 ± 3.1 | 0.20 |
| CRP in mg/dl | 1.2 ± 1.9 | 0.9 ± 1.4 | 0.37 |

**Table S4: Clinical and cellular characteristics of patients with progressive and non-progressive SARD-ILD, applying the original INBUILD criteria.** This table presents a detailed comparison of demographic and clinical data including HRCT pattern, extent of pulmonary disease, pulmonary function test results, and bronchoalveolar lavage fluid (BALF) cellular profiles, such as lymphocyte, neutrophil, and eosinophil proportions, between progressive and non-progressive cases based on the original INBUILD criteria. Based on the INBUILD criteria for disease progression, 17 patients (19.1%) were identified as progressors, while 72 (80.9%) were classified as non-progressors. Data are presented as mean ± standard deviation (SD) for continuous variables and as numbers (percentages) for categorical variables. Abbreviations: No./n, number; SARD-ILD, systemic autoimmune rheumatic disease-associated interstitial lung disease; BALF, bronchoalveolar lavage fluid; FVC (% predicted), forced vital capacity; DLCO (% predicted), diffusing capacity of the lungs for carbon monoxide; SD, standard deviation; HRCT, high-resolution computed tomography; NSIP, nonspecific interstitial pneumonia; UIP, usual interstitial pneumonia; OP, organizing pneumonia; LIP, lymphocytic interstitial pneumonia; BAL, bronchoalveolar lavage; CRP, C-reactive protein; G/L, giga per liter; mg/dl, milligram per deciliter. P-values <0.05 were considered statistically significant.

| Variable | Progressive | Non-Progressive | p-value |
|---|---|---|---|
| **No. of patients n (%)** | 17 (19.1) | 72 (80.9) | |
| **Male sex n (%)** | 5 (29.4) | 19 (26.4) | 1.00 |
| **Age (y, mean ± SD)** | 57.2 ± 15.9 | 57.5 ± 15.1 | 0.97 |
| **Disease duration (y, mean ± SD)** | 1.76 ± 2.80 | 1.92 ± 2.92 | 0.62 |
| **Ever-smoker n (%)** | 6 (35.3) | 37 (51.4) | 0.36 |
| **Immunosuppressive treatment n (%)** | 16 (94.1) | 59 (81.9) | 0.39 |
| **Antifibrotic treatment n (%)** | 2 (11.8) | 5 (6.9) | 0.62 |
| **Rituximab n (%)** | 2 (11.8) | 7 (9.7) | 0.68 |
| **Mycophenolate mofetil n (%)** | 7 (41.2) | 17 (23.6) | 0.24 |
| **Methotrexate n (%)** | 2 (11.8) | 9 (12.5) | 1.00 |
| **Leflunomide n (%)** | 0 (0.0) | 2 (2.8) | 1.00 |
| **Azathioprine n (%)** | 0 (0.0) | 2 (2.8) | 1.00 |
| **Tocilizumab n (%)** | 2 (11.8) | 3 (4.2) | 0.24 |
| **Glucocorticoids** | 6 (35.3) | 23 (31.9) | 1.00 |
| **FVC (% predicted, mean ± SD)** | 67.1 ± 23.5 | 75.8 ± 21.1 | 0.14 |
| **DLCO SB (%predicted, mean ± SD)** | 40.9 ± 16 | 51.4 ± 18.2 | 0.03 |
| **DLCO/VA (% predicted, mean ± SD)** | 62.6 ± 15.6 | 71.5 ± 17.0 | 0.06 |
| **SARD Diagnosis** | | | 0.07 |
| Myositis | 5 (29.4) | 10 (13.9) | 0.12 |
| Systemic Sclerosis | 4 (23.5) | 17 (23.6) | 0.99 |
| Rheumatoid Arthritis | 4 (23.5) | 11 (15.3) | 0.41 |
| Mixed connective tissue disease | 1 (5.9) | 22 (30.6) | 0.06 |
| Sjögren's syndrome | 3 (17.6) | 5 (6.9) | 0.18 |
| Others (IPAF, other) | 0 (0.0) | 7 (9.7) | 0.34 |
| **HRCT pattern n (%)** | | | 0.60 |
| NSIP | 9 (52.9) | 43 (59.7) | 0.61 |
| UIP | 2 (11.8) | 12 (16.7) | 0.62 |
| OP | 3 (17.6) | 3 (4.2) | 0.08 |
| LIP | 1 (5.9) | 3 (4.2) | 0.58 |
| Unclassified | 2 (11.8) | 8 (11.1) | 1.00 |
| Other | 0 (0.0) | 3 (4.2) | 1.00 |
| **Extent of disease on HRCT (%,mean ± SD)** | 27.1 ± 14.4 | 27.4 ± 19.3 | 0.76 |
| **BALF total cell count (cells/100ml, median, range)** | 14.2 ± 15.1 | 11.0 ± 10.6 | 0.52 |
| **BAL Neutrophils (%, mean ± SD, range)** | 8.73 ± 11.3 | 11.7 ± 19.2 | 0.87 |
| **BAL Lymphocytes (%, mean ± SD, range)** | 28.4 ± 28.0 | 18.2 ± 18.9 | **0.04** |

| | | | |
|---|---|---|---|
| **BAL Eosinophils (%, mean ± SD, range)** | 3.14 ± 3.1 | 3.12 ± 2.7 | 0.98 |
| **Peripheral blood parameters (mean ± SD)** | | | |
| Leukocytes (absolute in G/L) | 8.2 ± 3.4 | 7.8 ± 2.7 | 0.66 |
| Lymphocytes (absolute in G/L) | 1.2 ± 0.7 | 1.5 ± 0.6 | 0.10 |
| Lymphocytes (relative in %) | 15.5 ± 5.8 | 20.8 ± 9.3 | 0.03 |
| Monocytes (absolute in G/L) | 0.5 ± 0.2 | 0.6 ± 0.3 | 0.83 |
| Monocytes (relative in %) | 6.9 ± 2.4 | 7.9 ± 3.1 | 0.40 |
| CRP in mg/dl | 1.7 ± 2.3 | 0.8 ± 1.3 | 0.04 |

**Table S5: Multivariable logistic regression models for predicting disease progression in SARD-ILD.** This table presents the results of stepwise multivariable logistic regression models assessing the association between various clinical, pulmonary, radiographic, and therapeutic factors and the likelihood of disease progression in patients with SARD-ILD. Each column reflects the addition of individual variables starting from the baseline model, which includes age, FVC and extent of disease on HRCT. Odds ratios (OR) with 95% confidence intervals (CI) and p-values are reported for each variable in the respective models. The proportion of lymphocytes in BAL remained a significant predictor of disease progression across all models. Abbreviations: SARD-ILD, systemic autoimmune rheumatic disease-associated interstitial lung disease; BAL, bronchoalveolar lavage; FVC (% predicted), forced vital capacity; HRCT, high-resolution computed tomography; OR, odds ratio; CI, confidence interval; DLCO (% predicted), diffusing capacity of the lungs for carbon monoxide; UIP, usual interstitial pneumonia; NSIP, nonspecific interstitial pneumonia; OP, organizing pneumonia; LIP, lymphocytic interstitial pneumonia. P-values <0.05 were considered statistically significant, and significant values are highlighted in bold.

| | **Model 1 (Baseline)** | | **Model 2 (+Sex)** | | **Model 3 (+Disease duration)** | | **Model 4 (+Treatment)** | | **Model 5 (+DLCO)** | | **Model 6 (+Diagnosis)** | | **Model 7 (+HRCT pattern)** | | **Model 8 (all with diagnosis)** | |
|---|---|---|---|---|---|---|---|---|---|---|---|---|---|---|---|---|
| **Variable** | **OR (95% CI)** | **p-value** | **OR (95% CI)** | **p-value** | **OR (95% CI)** | **p-value** | **OR (95% CI)** | **p-value** | **OR (95% CI)** | **p-value** | **OR (95% CI)** | **p-value** | **OR (95% CI)** | **p-value** | **OR (95% CI)** | **p-value** |
| **Age (years)** | 1.04 (1.01-1.09) | **0.03** | 1.04 (1.00-1.09) | **0.03** | 1.04 (1.00-1.09) | **0.04** | 1.05 (1.05-1.09) | **0.03** | 1.06 (1.02-1.12) | **0.01** | 1.06 (1.01-1.12) | **0.04** | 1.05 (1.01-1.10) | **0.03** | 1.11 (1.03-1.21) | **0.01** |
| **FVC (% predicted)** | 0.98 (0.95-1.01) | 0.16 | 0.98 (0.95-1.01) | 0.16 | 0.98 (0.95-1.01) | 0.18 | 0.98 (0.94-1.01) | 0.12 | 0.99 (0.95-1.02) | 0.40 | 0.97 (0.94-1.01) | 0.13 | 0.98 (0.94-1.01) | 0.15 | 0.98 (0.93-1.02) | 0.26 |
| **Total disease extent on HRCT (%)** | 0.99 (0.96-1.03) | 0.73 | 0.99 (0.96-1.02) | 0.68 | 1.00 (0.96-1.03) | 0.77 | 1.00 (0.97-1.03) | 0.92 | 0.98 (0.94-1.02) | 0.36 | 1.00 (0.97-1.04) | 0.88 | 0.99 (0.96-1.02) | 0.57 | 0.98 (0.93-1.04) | 0.51 |
| **Lymphocyte proportion (%)** | **1.05 (1.02-1.07)** | **<0.01** | **1.05 (1.02-1.07)** | **<0.01** | **1.04 (1.02-1.07)** | **<0.01** | **1.05 (1.02-1.08)** | **<0.01** | **1.06 (1.03-1.09)** | **<0.01** | **1.06 (1.02-1.09)** | **<0.01** | **1.04 (1.01-1.07)** | **0.01** | **1.08 (1.04-1.14)** | **<0.01** |
| **Sex (female)** | - | - | 0.65 (0.22-1.97) | 0.44 | - | - | - | - | - | - | - | - | - | - | 0.79 (0.16-3.81) | 0.77 |

| | | | | | | | | | | | | | | | | |
|---|---|---|---|---|---|---|---|---|---|---|---|---|---|---|---|---|
| **Disease duration (years)** | - | - | - | - | 0.98 (0.80-1.18) | 0.85 | - | - | - | - | - | - | - | - | 1.12 (0.87-1.45) | 0.39 |
| **Treatment** | | | | | | | | | | | | | | | | |
| | **Model 1 (Baseline)** | | **Model 2 (+Sex)** | | **Model 3 (+Disease duration)** | | **Model 4 (+Treatment)** | | **Model 5 (+DLCO)** | | **Model 6 (+Diagnosis)** | | **Model 7 (+HRCT pattern)** | | **Model 8 (all with diagnosis)** | |
| Rituximab (yes) | - | - | - | - | - | - | 0.55 (0.05-3.52) | 0.57 | - | - | - | - | - | - | 0.45 (0.02-6.03) | 0.57 |
| Mycofenolate mofetil (yes) | - | - | - | - | - | - | 1.05 (0.28-3.74) | 0.94 | - | - | - | - | - | - | 0.93 (0.17-5.30) | 0.94 |
| Glucocorticoids (yes) | - | - | - | - | - | - | 0.64 (0.16-2.24) | 0.51 | - | - | - | - | - | - | 0.32 (0.04-2.14) | 0.26 |
| Antifibrotic (yes) | - | - | - | - | - | - | 0.58 (0.06-3.91) | 0.59 | - | - | - | - | - | - | 0.80 (0.05-11.73) | 0.87 |
| **DLCO (% predicted)** | - | - | - | - | - | - | - | - | 0.97 (0.93-1.01) | 0.22 | - | - | - | - | 0.98 (0.92-1.03) | 0.46 |
| **SARD Diagnosis (Reference: Myositis)** | | | | | | | | | | | | | | | | |

| | | | | | | | | | | | | | | | | |
|---|---|---|---|---|---|---|---|---|---|---|---|---|---|---|---|---|
| Systemic Sclerosis | - | - | - | - | - | - | - | - | - | - | 2.21 (0.39-14.39) | 0.38 | - | - | 3.55 (0.41-37.46) | 0.26 |
| Rheumatoid Arthritis | - | - | - | - | - | - | - | - | - | - | 0.60 (0.07-4.63) | 0.63 | - | - | 0.23 (0.02-2.83) | 0.26 |
| | **Model 1 (Baseline)** | | **Model 2 (+Sex)** | | **Model 3 (+Disease duration)** | | **Model 4 (+Treatment)** | | **Model 5 (+DLCO)** | | **Model 6 (+Diagnosis)** | | **Model 7 (+HRCT pattern)** | | **Model 8 (all with diagnosis)** | |
| Mixed Connective Tissue Disease | - | - | - | - | - | - | - | - | - | - | **0.10 (0.01-0.74)** | **0.03** | - | - | 0.21 (0.02-1.97) | 0.19 |
| Sjögren’s Syndrome | - | - | - | - | - | - | - | - | | | 1.74 (0.14-27.09) | 0.67 | - | - | 1.15 (0.07-23.57) | 0.92 |
| Other | - | - | - | - | - | - | - | - | | | 1.59 (0.16-16.03) | 0.68 | - | - | 1.09 (0.06-21.69) | 0.95 |
| **CT pattern (Reference: NSIP)** | | | | | | | | | | | | | | | | |
| LIP | - | - | - | - | - | - | - | - | - | - | - | - | 0.52 (0.01-17.83) | 0.70 | - | - |
| OP | - | - | - | - | - | - | - | - | - | - | - | - | 2.81 (0.19-44.31) | 0.44 | - | - |

| | | | | | | | | | | | | | | | | | |
|---|---|---|---|---|---|---|---|---|---|---|---|---|---|---|---|---|---|
| | UIP | - | - | - | - | - | - | - | - | - | - | - | - | 0.72 (0.16-2.91) | 0.65 | - | - |
| | Unclassified | - | - | - | - | - | - | - | - | - | - | - | - | 1.72 (0.33-9.02) | 0.51 | - | - |
| | Other | - | - | - | - | - | - | - | - | - | - | - | - | 0.00 | 0.99 | - | - |

**Table S6: Clinical and cellular characteristics of patients with SARD-ILD stratified by BAL lymphocyte proportion (≤ 9% vs. > 9%).** This table presents a detailed comparison of demographic and clinical data including HRCT pattern, extent of pulmonary disease, pulmonary function test results, and bronchoalveolar lavage fluid (BALF) cellular profiles, such as lymphocyte, neutrophil, and eosinophil proportions, based on the identified cut-off of 9% BAL lymphocytes. Data are presented as mean ± standard deviation (SD) for continuous variables and as numbers (percentages) for categorical variables. Abbreviations: No./n, number; SARD-ILD, systemic autoimmune rheumatic disease-associated interstitial lung disease; BALF, bronchoalveolar lavage fluid; FVC (% predicted), forced vital capacity; DLCO (% predicted), diffusing capacity of the lungs for carbon monoxide; SD, standard deviation; HRCT, high-resolution computed tomography; NSIP, nonspecific interstitial pneumonia; UIP, usual interstitial pneumonia; OP, organizing pneumonia; LIP, lymphocytic interstitial pneumonia; BAL, bronchoalveolar lavage; CRP, C-reactive protein; G/L, giga per liter; mg/dl, milligram per deciliter. P-values <0.05 were considered statistically significant.

| Variable | BAL Lymphocyte % ≤ 9 | BAL Lymphocyte % > 9 | p-value |
|---|---|---|---|
| **No. of patients n (%)** | 42 (47.2) | 47 (52.8) | |
| **Male sex n (%)** | 7 (16.7) | 17 (36.1) | 0.07 |
| **Age (y, mean ± SD)** | 57.8 ± 15.5 | 57.1 ± 15.0 | 0.85 |
| **Disease duration (y, mean ± SD)** | 2.0 ± 2.5 | 1.8 ± 3.2 | 0.24 |
| **Ever-smoker n (%)** | 23 (54.8) | 20 (42.6) | 0.25 |
| **Immunosuppressive treatment n (%)** | 36 (85.7) | 39 (83.0) | 0.95 |
| **Antifibrotic treatment n (%)** | 4 (9.5) | 3 (6.4) | 0.70 |
| **Rituximab n (%)** | 7 (16.7) | 2 (4.3) | 0.08 |
| **Mycophenolate mofetil n (%)** | 10 (23.8) | 14 (29.8) | 0.69 |
| **Methotrexate n (%)** | 6 (14.3) | 5 (10.6) | 0.75 |
| **Leflunomide n (%)** | 0 (0.0) | 2 (4.3) | 0.50 |
| **Azathioprine n (%)** | 1 (2.4) | 1 (2.1) | 1.00 |
| **Tocilizumab n (%)** | 1 (2.4) | 4 (8.5) | 1.00 |
| **Glucocorticoids n (%)** | 12 (28.6) | 17 (36.2) | 0.59 |
| **FVC (% predicted, mean ± SD)** | 76.6 ± 23.9 | 71.9 ± 19.6 | 0.45 |
| **DLCO SB (% predicted, mean ± SD)** | 50.7 ± 17.9 | 48.4 ± 18.6 | 0.51 |
| **DLCO/VA (% predicted, mean ± SD)** | 69.7 ± 17.7 | 70 ± 16.6 | 0.85 |
| **SARD Diagnosis** | | | 0.09 |
| Myositis | 5 (11.9) | 10 (21.3) | 0.27 |
| Systemic Sclerosis | 9 (21.4) | 12 (25.5) | 0.80 |
| Rheumatoid Arthritis | 6 (14.3) | 9 (19.1) | 0.58 |
| Mixed connective tissue disease | 17 (40.5) | 6 (12.8) | <0.01 |
| Sjögren's syndrome | 3 (7.1) | 5 (10.6) | 0.72 |
| Others (IPAF, other) | 2 (4.8) | 5 (10.6) | 0.44 |
| **HRCT pattern n (%)** | | | 0.05 |
| NSIP | 1 (2.4) | 3 (6.4) | 0.62 |
| UIP | 0 (0.0) | 6 (12.8) | 0.03 |
| OP | 30 (71.4) | 22 (46.8) | 0.02 |
| LIP | 3 (7.1) | 7 (14.9) | 0.32 |
| Unclassified | 2 (4.8) | 1 (2.1) | 0.60 |
| Other | 6 (14.3) | 8 (17.0) | 0.78 |
| **Extent of disease on HRCT (%,mean ± SD)** | 26.6 ± 17.8 | 28.0 ± 19.1 | 0.83 |
| **BALF total cell count (cells/100ml, median, range)** | 11.8 ± 12.3 | 11.4 ± 11.0 | 0.85 |
| **BAL Neutrophils (%, mean ± SD, range)** | 13.3 ± 22.9 | 9.46 ± 13.2 | 0.59 |
| **BAL Lymphocytes (%, mean ± SD, range)** | 5.6 ± 2.6 | 33.1 ± 22.1 | <0.01 |
| **BAL Eosinophils (%, mean ± SD, range)** | 2.9 ± 2.5 | 3.3 ± 2.9 | 0.70 |
| **Peripheral blood parameters (mean ± SD)** | | | |
| Leukocytes (absolute in G/L) | 8.0 ± 3.0 | 7.8 ± 2.8 | 0.66 |
| Lymphocytes (absolute in G/L) | 1.5 ± 0.7 | 1.5 ± 0.7 | 0.94 |
| Lymphocytes (relative in %) | 19.6 ± 9.0 | 20.0 ± 9.1 | 0.61 |
| Monocytes (absolute in G/L) | 0.6 ± 0.3 | 0.5 ± 0.2 | 0.09 |
| Monocytes (relative in %) | 8.4 ± 3.4 | 7.1 ± 2.5 | 0.13 |
| CRP in mg/dl | 1.0 ± 1.5 | 1.0 ± 1.6 | 0.90 |

**Table S7: Multivariable logistic regression analysis including eosinophil proportion in BAL for predicting disease progression in SARD-ILD.** This table presents the results of a multivariable logistic regression model assessing the association between age, FVC, radiographic disease extent, and eosinophil proportion in BAL for predicting disease progression in patients with SARD-ILD. Odds ratios (OR) with 95% confidence intervals (CI) and p-values are reported for each variable. Abbreviations: SARD-ILD, systemic autoimmune rheumatic disease-associated interstitial lung disease; FVC (%predicted), forced vital capacity; HRCT, high-resolution computed tomography; OR, odds ratio; CI, confidence interval.

| | **Model** | |
|---|---|---|
| **Variable** | **OR (95% CI)** | **p-value** |
| **Age (years)** | 1.05 (0.99-1.11) | 0.11 |
| **FVC (% predicted)** | 1.00 (0.96-1.04) | 0.88 |
| **Total disease extent on HRCT (%)** | 1.00 (0.96-1.04) | 1.00 |
| **Eosinophil proportion (%)** | 1.00 (0.77-1.28) | 0.97 |

**Table S8: Multivariable logistic regression analysis including neutrophil proportion in BAL for predicting disease progression in SARD-ILD.** This table presents the results of a multivariable logistic regression model assessing the association between age, FVC, radiographic disease extent, and neutrophil proportion in BAL for predicting disease progression in patients with SARD-ILD. Odds ratios (OR) with 95% confidence intervals (CI) and p-values are reported for each variable. Abbreviations: SARD-ILD, systemic autoimmune rheumatic disease-associated interstitial lung disease; BAL, bronchoalveolar lavage; FVC (%predicted), forced vital capacity; HRCT, high-resolution computed tomography; OR, odds ratio; CI, confidence interval.

| | **Model** | |
|---|---|---|
| **Variable** | **OR (95% CI)** | **p-value** |
| **Age (years)** | 1.02 (0.97-1.07) | 0.43 |
| **FVC (% predicted)** | 0.98 (0.95-1.01) | 0.26 |
| **Total disease extent on HRCT (%)** | 0.99 (0.96-1.03) | 0.68 |
| **Neutrophil proportion (%)** | 0.99 (0.94-1.02) | 0.44 |

**Table S9: Multivariable logistic regression analysis including peripheral blood parameters and BAL lymphocyte proportion for predicting disease progression in SARD-ILD.** This table presents the results of a multivariable logistic regression model evaluating the association between age, FVC, radiographic disease extent, lymphocyte proportion in BAL (=baseline model), and peripheral blood parameters (CRP, leukocytes, monocytes, lymphocytes) for predicting disease progression in patients with SARD-ILD. Odds ratios (OR) with 95% confidence intervals (CI) and p-values are reported for each variable. P-values <0.05 were considered statistically significant, and significant values are highlighted in bold. Abbreviations: SARD-ILD, systemic autoimmune rheumatic disease-associated interstitial lung disease; BAL, bronchoalveolar lavage; FVC (% predicted), forced vital capacity; HRCT, high-resolution computed tomography; CRP, C-reactive protein; OR, odds ratio; CI, confidence interval.

| | **Model** | |
|---|---|---|
| **Variable** | **OR (95% CI)** | **p-value** |
| **Age (years)** | 1.04 (1.00-1.09) | 0.07 |
| **FVC (% predicted)** | 0.99 (0.95-1.02) | 0.40 |
| **Total disease extent on HRCT (%)** | 1.00 (0.96-1.03) | 0.89 |
| **Lymphocyte proportion (%) (BAL)** | **1.04 (1.02-1.07)** | **<0.01** |
| **Leukocytes (blood)** | 1.00 (0.80-1.24) | 1.00 |
| **Monocytes (blood)** | 0.20 (0.01-2.82) | 0.26 |
| **Lymphocytes (blood)** | 1.02 (0.41-2.54) | 0.96 |
| **C-reactive protein (blood)** | 1.08 (0.77-1.47) | 0.64 |

**Table S10. Multivariable logistic regression analysis for the progression endpoint of INBUILD, ILD-related mortality, or lung transplantation, excluding treatment escalation.** Odds ratios (OR) with 95% confidence intervals (CI) and p-values are shown. Abbreviations: SARD-ILD, systemic autoimmune rheumatic disease-associated interstitial lung disease; FVC (% predicted), forced vital capacity; DLCO (% predicted), diffusion capacity of the lungs for carbon monoxide; HRCT, high-resolution computed tomography; CRP, C-reactive protein; OR, odds ratio; CI, confidence interval.

| | Variable | OR | 95% CI lower | 95% CI upper | p-value |
|---|---|---|---|---|---|
| **Age (years)** | | 1.03 | 0.97 | 1.12 | 0.32 |
| **FVC (% predicted)** | | 1.00 | 0.96 | 1.04 | 0.93 |
| **Total disease extent on HRCT (%)** | | 0.99 | 0.94 | 1.04 | 0.78 |
| **Lymphocyte proportion (%)** | | 1.04 | 1.00 | 1.1 | 0.03 |
| **Sex (male)** | | 0.45 | 0.10 | 2.1 | 0.31 |
| **Disease duration (years)** | | 0.97 | 0.71 | 1.26 | 0.87 |
| **Treatment (yes)** | | | | | |
| | **Rituximab** | 1.67 | 0.10 | 22.52 | 0.70 |
| | **Mycofenolate mofetil** | 2.16 | 0.36 | 13.88 | 0.39 |
| | **Glucocorticoids** | 0.39 | 0.05 | 2.38 | 0.33 |
| | **Antifibrotic** | 1.89 | 0.12 | 28.93 | 0.64 |
| **DLCO (% predicted)** | | 0.95 | 0.88 | 1.00 | 0.10 |
| **SARD-Diagnosis (Reference: Myositis)** | | | | | |
| | **Systemic Sclerosis** | 1.05 | 0.11 | 10.24 | 0.96 |
| | **Rheumatoid Arthritis** | 1.04 | 0.09 | 13.48 | 0.97 |
| | **Mixed Connective Tissue Disease** | 0.22 | 0.01 | 2.37 | 0.24 |
| | **Sjögren's Syndrome** | 1.50 | 0.12 | 19.88 | 0.75 |
| | **Other** | 0.75 | 0.04 | 13.63 | 0.84 |
| | | | | | |
| Pseudo-$R^2$ = 47.4% | | | | | |
| AIC = 96.7 | | | | | |

**Table S11: Subgroup and interaction analyses evaluating the association between bronchoalveolar lavage (BAL) lymphocyte proportion and disease progression according to BAL indication at baseline.** The association between BAL lymphocyte proportion and disease progression remained significant in patients undergoing BAL during diagnostic evaluation of newly diagnosed ILD and in patients with pre-existing/progressive ILD. No significant interaction between BAL indication and BAL lymphocyte proportion was observed. Abbreviations: BAL, bronchoalveolar lavage; ILD, interstitial lung disease; OR, odds ratio; CI, confidence interval.

| BAL indication subgroup | OR for BAL lymphocyte proportion | 95% CI | p-value |
|---|---|---|---|
| Newly diagnosed ILD | 1.05 | 1.02-1.10 | 0.01 |
| Pre-existing/progressive ILD | 1.04 | 1.00-1.08 | 0.04 |
| Interaction term | - | - | 0.55 |

**Table S12: Multivariable Firth penalized logistic regression analyses evaluating the association between bronchoalveolar lavage (BAL) lymphocyte proportion and disease progression across three different progression endpoints, including original INBUILD progression criteria, the endpoint consisting of INBUILD progression, ILD-related mortality or lung transplantation, and the composite progression endpoint including treatment escalation**. Penalized regression analyses were performed to reduce small-sample bias and potential model overfitting related to the limited number of progression events. Abbreviations: BAL, bronchoalveolar lavage; ILD, interstitial lung disease; HRCT, high-resolution computed tomography; DLCO, diffusion capacity for carbon monoxide; OR, odds ratio; CI, confidence interval; LTX, lung transplantation.

| | INBUILD | | | | INBUILD + death/LTX | | | | Composite endpoint | | | |
|---|---|---|---|---|---|---|---|---|---|---|---|---|
| Variable | OR | 95% CI lower | 95% CI upper | p-value | OR | 95% CI lower | 95% CI upper | p-value | OR | 95% CI lower | 95% CI upper | p-value |
| **Age (years)** | 1.02 | 0.96 | 1.1 | 0.47 | 1.03 | 0.97 | 1.10 | 0.38 | 1.08 | 1.02 | 1.16 | 0.01 |
| **FVC (% predicted)** | 1.00 | 0.96 | 1.03 | 0.82 | 1.00 | 0.96 | 1.04 | 0.96 | 0.98 | 0.95 | 1.02 | 0.31 |
| **Total disease extent on HRCT (%)** | 0.97 | 0.92 | 1.02 | 0.28 | 1.00 | 0.95 | 1.04 | 0.89 | 0.99 | 0.94 | 1.04 | 0.62 |
| **Lymphocyte proportion (%)** | 1.04 | 1.00 | 1.08 | 0.04 | 1.03 | 1.00 | 1.07 | 0.04 | 1.06 | 1.02 | 1.10 | <0.01 |
| Sex (male) | 0.94 | 0.22 | 4.27 | 0.94 | 0.58 | 0.15 | 2.16 | 0.42 | 0.84 | 0.22 | 3.14 | 0.79 |
| Disease duration (years) | 1.05 | 0.82 | 1.31 | 0.68 | 1.00 | 0.77 | 1.23 | 0.99 | 1.09 | 0.88 | 1.35 | 0.42 |
| Treatment (yes) | | | | | | | | | | | | |
| Rituximab | 1.32 | 0.13 | 11.19 | 0.80 | 1.47 | 0.16 | 11.73 | 0.71 | 0.57 | 0.05 | 4.70 | 0.61 |
| Mycofenolate mofetil | 2.58 | 0.54 | 14.59 | 0.23 | 1.82 | 0.40 | 8.41 | 0.43 | 1.01 | 0.23 | 4.42 | 0.99 |
| Glucocorticoids | 0.41 | 0.07 | 1.91 | 0.26 | 0.54 | 0.10 | 2.40 | 0.43 | 0.48 | 0.08 | 2.27 | 0.36 |
| Antifibrotic | 3.45 | 0.32 | 44.11 | 0.30 | 1.64 | 0.16 | 15.87 | 0.67 | 0.86 | 0.08 | 8.19 | 0.90 |
| DLCO (% predicted) | 0.96 | 0.9 | 1.01 | 0.11 | 0.97 | 0.91 | 1.01 | 0.15 | 0.99 | 0.93 | 1.03 | 0.60 |
| SARD-Diagnosis (Reference: Myositis) | | | | | | | | | | | | |
| Systemic Sclerosis | 0.86 | 0.12 | 5.88 | 0.87 | 1.01 | 0.15 | 6.81 | 0.99 | 2.45 | 0.41 | 17.50 | 0.33 |
| Rheumatoid Arthritis | 0.86 | 0.09 | 8.04 | 0.89 | 1.06 | 0.13 | 9.15 | 0.96 | 0.32 | 0.04 | 2.71 | 0.30 |
| Mixed Connective Tissue Disease | 0.28 | 0.02 | 2.13 | 0.22 | 0.36 | 0.03 | 2.52 | 0.31 | 0.34 | 0.04 | 2.14 | 0.25 |
| Sjögren's Syndrome | 1.36 | 0.15 | 12.98 | 0.78 | 1.45 | 0.17 | 12.72 | 0.73 | 1.13 | 0.11 | 13.17 | 0.92 |
| Other | 0.14 | 0.00 | 2.9 | 0.22 | 0.88 | 0.07 | 10.23 | 0.92 | 1.08 | 0.09 | 13.34 | 0.95 |

**Table S13: Multivariable logistic regression analysis including BAL lymphocyte proportion >9% for predicting disease progression in SARD-ILD.** This table presents the results of a multivariable logistic regression analysis evaluating the association between age, FVC (% predicted), total disease extent on HRCT, and BAL lymphocyte proportion >9% with disease progression in patients with SARD-ILD. Odds ratios (OR) with 95% confidence intervals (CI) and p-values are reported for each variable. P-values <0.05 were considered statistically significant, and significant values are highlighted in bold. Abbreviations: SARD-ILD, systemic autoimmune rheumatic disease-associated interstitial lung disease; BAL, bronchoalveolar lavage; FVC (% predicted), forced vital capacity; HRCT, high-resolution computed tomography; OR, odds ratio; CI, confidence interval.

| | Model | |
|---|---|---|
| **Variable** | **OR (95% CI)** | **p-value** |
| **Age (years)** | 1.04 (1.00-1.09) | 0.06 |
| **FVC (% predicted)** | 0.98 (0.94-1.01) | 0.14 |
| **Total disease extent on HRCT (%)** | 0.99 (0.96-1.02) | 0.47 |
| **Lymphocyte proportion >9%** | **13.14 (4.20-51.98)** | **<0.01** |

**Table S14: Multivariable logistic regression analysis including BAL lymphocyte proportion >15% for predicting disease progression in SARD-ILD.** This table presents the results of a multivariable logistic regression analysis evaluating the association between age, FVC (% predicted), total disease extent on HRCT, and BAL lymphocyte proportion >15% with disease progression in patients with SARD-ILD. Odds ratios (OR) with 95% confidence intervals (CI) and p-values are reported for each variable. P-values <0.05 were considered statistically significant, and significant values are highlighted in bold. Abbreviations: SARD-ILD, systemic autoimmune rheumatic disease-associated interstitial lung disease; BAL, bronchoalveolar lavage; FVC (% predicted), forced vital capacity; HRCT, high-resolution computed tomography; OR, odds ratio; CI, confidence interval.

| | Model | |
|---|---|---|
| **Variable** | **OR (95% CI)** | **p-value** |
| **Age (years)** | 1.05 (1.01-1.10) | 0.02 |
| **FVC (% predicted)** | 0.98 (0.94-1.01) | 0.12 |
| **Total disease extent on HRCT (%)** | 1.00 (0.97-1.03) | 0.93 |
| **Lymphocyte proportion >15%** | **9.02 (3.07-30.59)** | **<0.01** |

## Supplementary Figures

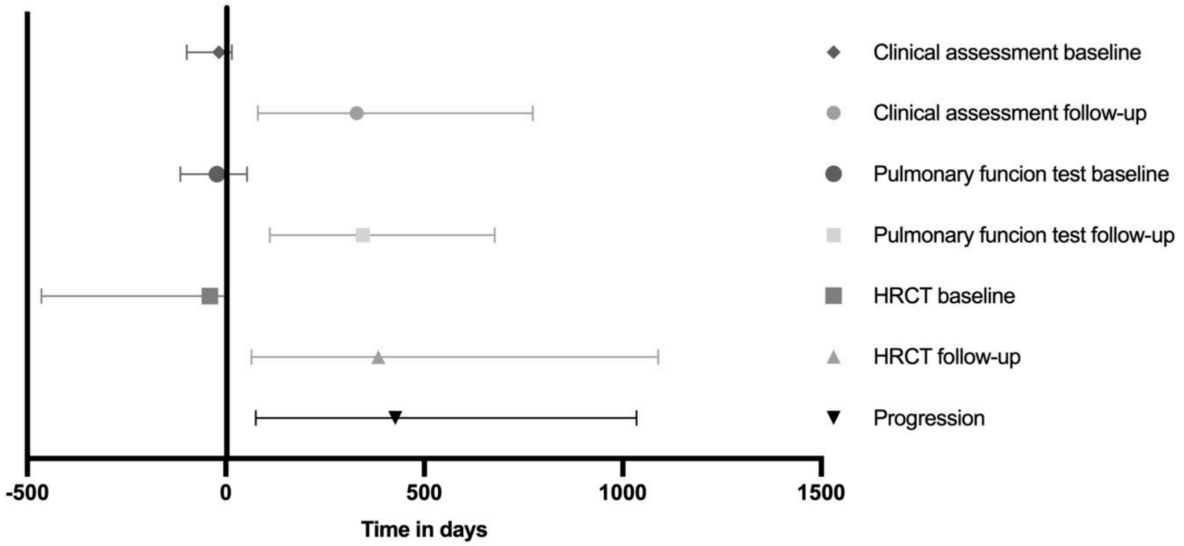


**Figure S1: Timeline of assessments.** This figure illustrates the timing of baseline and follow-up assessments, including clinical evaluations, pulmonary function tests, and HRCT imaging, as well as the recorded time point of disease progression. Day 0 represents the time of bronchoscopy with BALF sampling. Data are presented as median values with 95% confidence intervals. Abbreviation: HRCT, high-resolution computed tomography

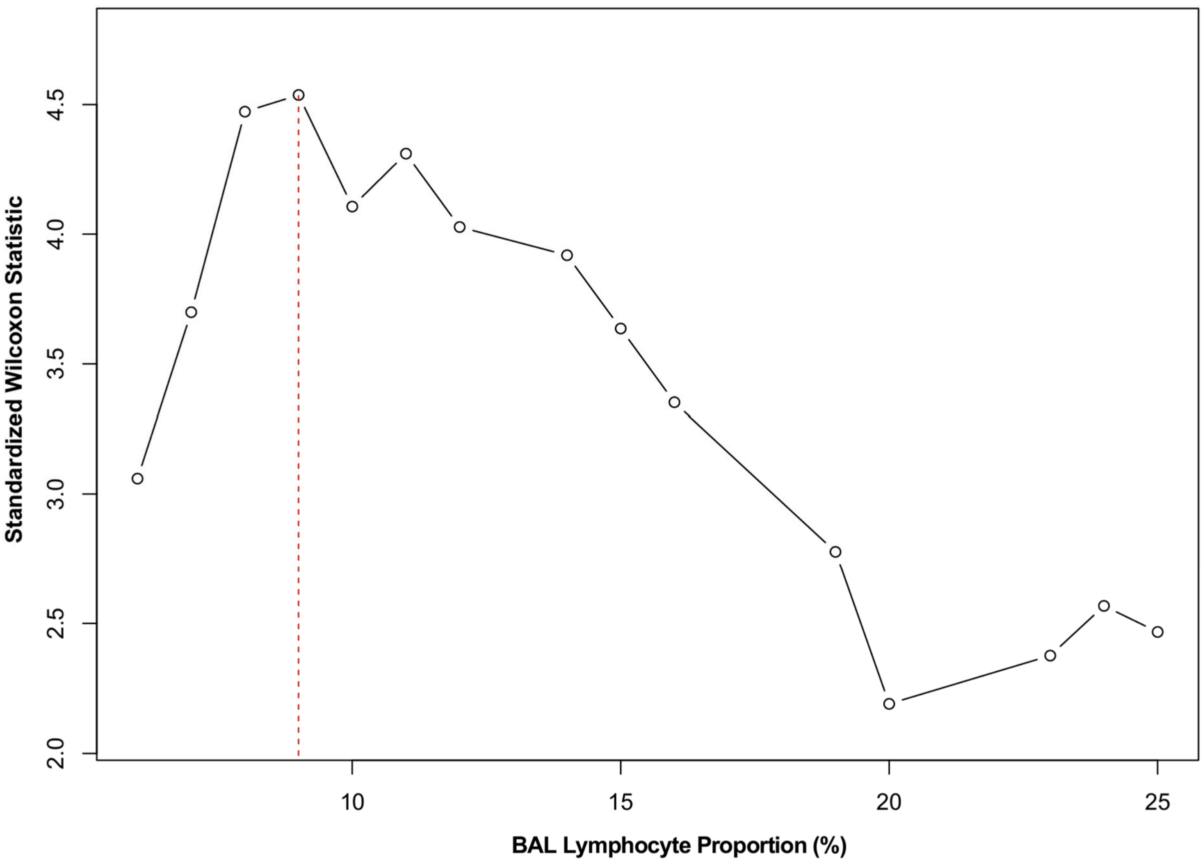


**Figure S2: Standardized Wilcoxon Statistic for identifying optimal cutoff of BAL lymphocyte proportion.** This figure displays the standardized Wilcoxon statistic, used to determine the optimal binary threshold of BAL lymphocyte proportion for distinguishing between progressive and non-progressive SARD-ILD. The analysis identified a threshold of 9% as the most effective in distinguishing disease progression.

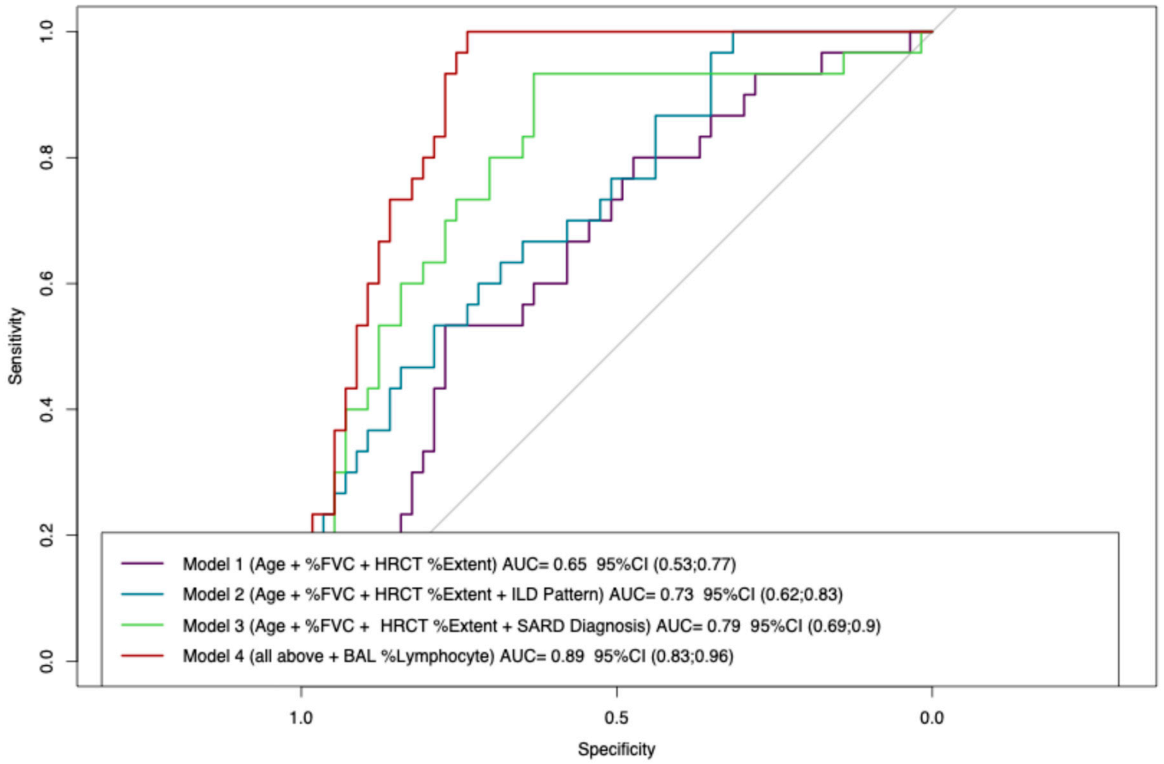


**Figure S3: Receiver operating characteristic (ROC) models comparing prediction of disease progression using clinical parameters alone versus with BAL lymphocyte proportion.** The addition of BAL lymphocyte proportion in the final step (Model 4) resulted in the highest predictive accuracy forf identifying patients at risk for progression Abbreviations: SARD, systemic autoimmune rheumatic disease; ILD interstitial lung disease; BAL, bronchoalveolar lavage; FVC (% predicted), forced vital capacity; HRCT, high-resolution computed tomography; %, proportion/extent; AUC, area under the curve; CI, confidence interval.

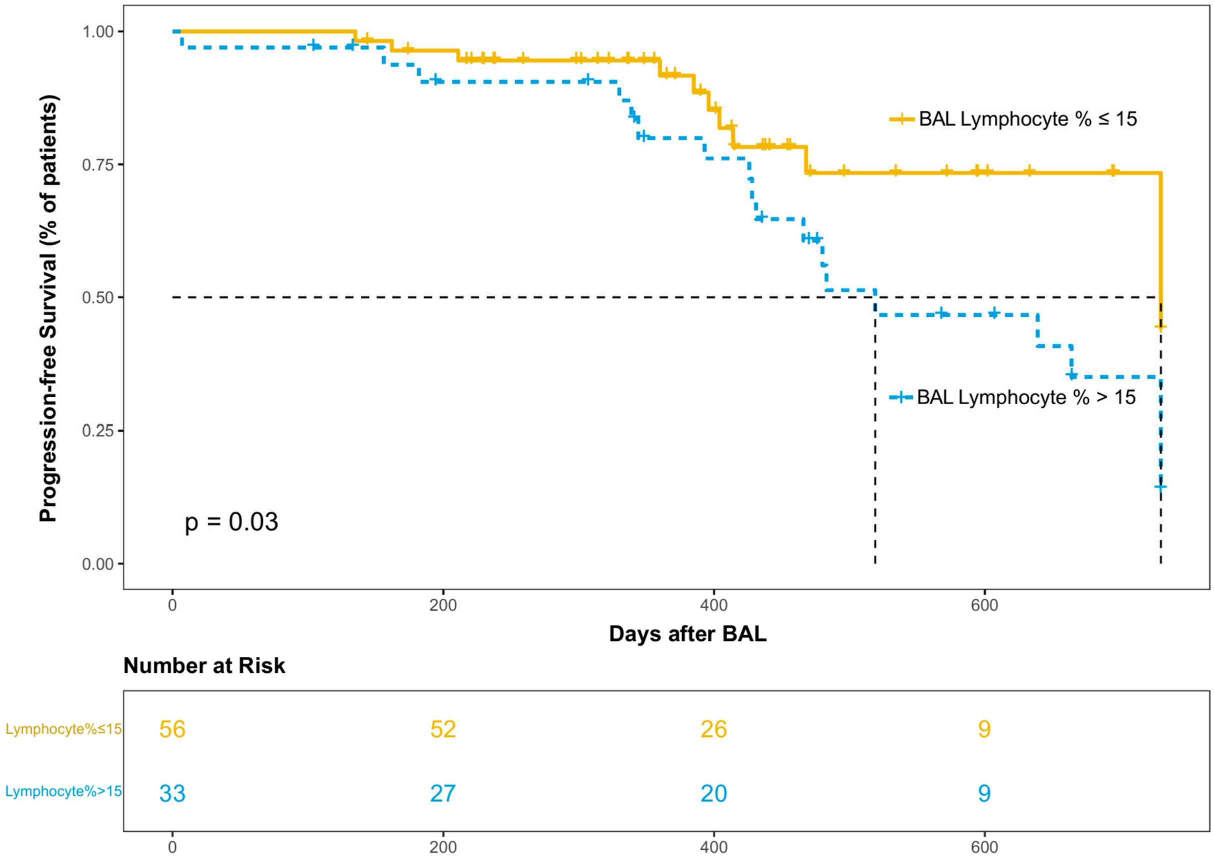


**Figure S4: Kaplan-Meier analysis for BAL lymphocyte proportion in predicting disease progression.** We stratified patients by BAL lymphocyte proportion (>15% vs. ≤15%) and demonstrates a significant difference in progression-free survival (p=0.03). SARD-ILD patients with a lymphocyte proportion >15% had a median progression-free survival of 519 days, while those with ≤15% 740 days. Abbreviations: BAL, bronchoalveolar lavage; SARD-ILD, systemic autoimmune rheumatic disease-associated interstitial lung disease; %, proportion.

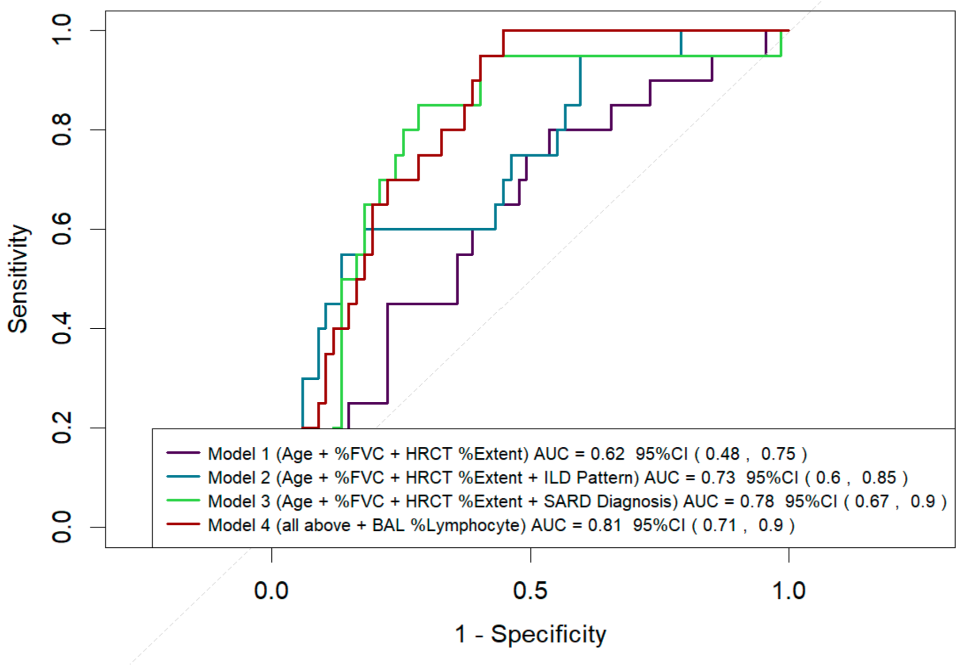


**Figure S5: Receiver operating characteristic (ROC) models comparing prediction of disease progression using clinical parameters alone vs with BAL lymphocyte proportion. Progression was defined as original INBUILD progression, ILD-related mortality, or lung transplantation, excluding treatment escalation.** Abbreviations: SARD, systemic autoimmune rheumatic disease; ILD, interstitial lung disease; BAL, bronchoalveolar lavage; FVC (% predicted), forced vital capacity; HRCT, high-resolution computed tomography; %, proportion/extent; AUC, area under the curve; CI, confidence interval.

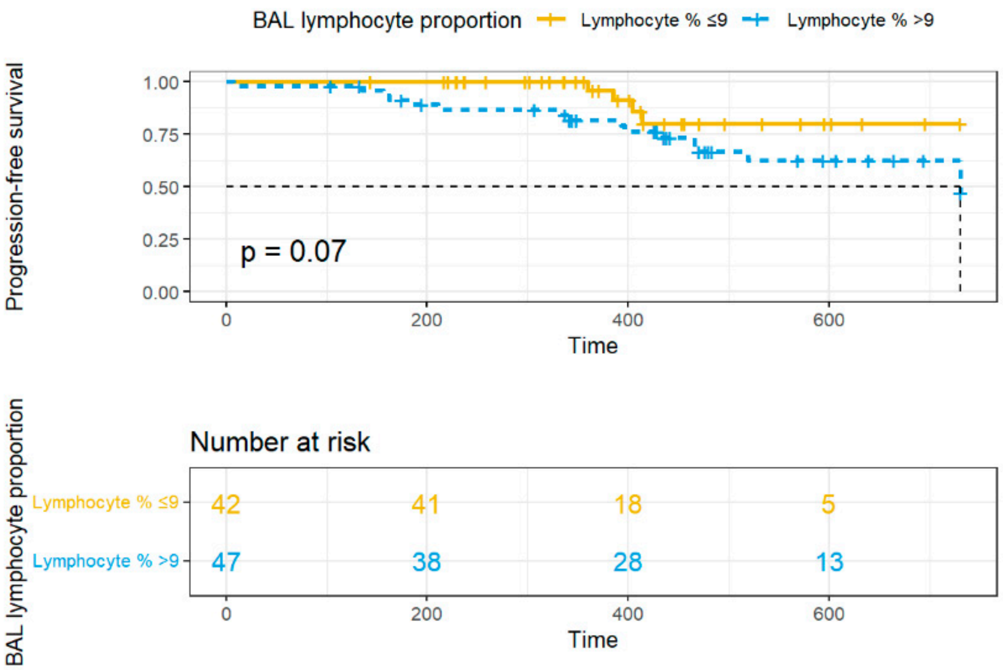


**Figure S6: Kaplan-Meier analysis for progression-free survival according to bronchoalveolar lavage (BAL) lymphocyte proportion using a cut-off of 9. Progression was defined using the objective composite endpoint consisting of original INBUILD progression criteria, lung transplantation, or ILD-related mortality.** Abbreviations: BAL, bronchoalveolar lavage; ILD, interstitial lung disease.

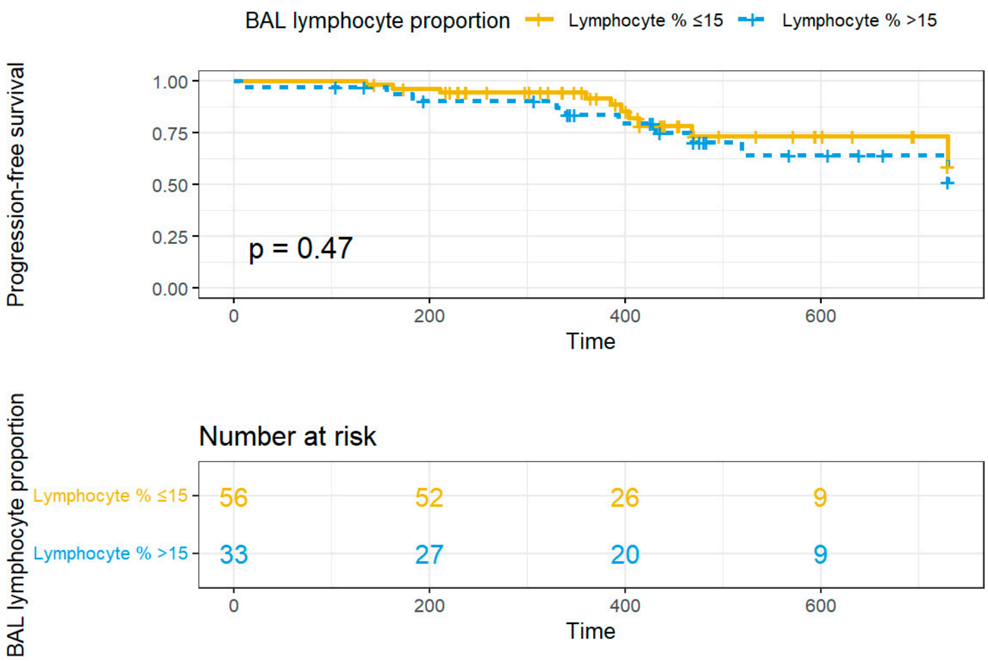


**Figure S7: Kaplan-Meier analysis for progression-free survival according to bronchoalveolar lavage (BAL) lymphocyte proportion using a cut-off of 15%. Progression was defined using the objective composite endpoint consisting of original INBUILD progression criteria, lung transplantation, or ILD-related mortality**. Abbreviations: BAL, bronchoalveolar lavage; ILD, interstitial lung disease.

## Supplementary References

1. Oldham JM, Lee CT, Wu Z, Bowman WS, Pugashetti JV, Dao N, et al. Lung function trajectory in progressive fibrosing interstitial lung disease. Eur Respir J. 2022;59(6).
2. Goh NS, Desai SR, Veeraraghavan S, Hansell DM, Copley SJ, Maher TM, et al. Interstitial lung disease in systemic sclerosis: a simple staging system. Am J Respir Crit Care Med. 2008;177(11):1248-54.
3. Hansell DM, Bankier AA, MacMahon H, McLoud TC, Müller NL, Remy J. Fleischner Society: glossary of terms for thoracic imaging. Radiology. 2008;246(3):697-722.
